# Competing tradeoffs between increasing marine mammal predation and fisheries harvest of Chinook salmon

**DOI:** 10.1038/s41598-017-14984-8

**Published:** 2017-11-20

**Authors:** Brandon E. Chasco, Isaac C. Kaplan, Austen C. Thomas, Alejandro Acevedo-Gutiérrez, Dawn P. Noren, Michael J. Ford, M. Bradley Hanson, Jonathan J. Scordino, Steven J. Jeffries, Kristin N. Marshall, Andrew O. Shelton, Craig Matkin, Brian J. Burke, Eric J. Ward

**Affiliations:** 1 Contractor to Conservation Biology Division, NOAA NMFS Northwest Fisheries Science Center, National Marine Fisheries Service, National Oceanic and Atmospheric Administration, 2725, Montlake Blvd. East, Seattle, WA 98112 USA; 2 Conservation Biology Division, NOAA NMFS Northwest Fisheries Science Center, National Marine Fisheries Service, National Oceanic and Atmospheric Administration, 2725, Montlake Blvd. East, Seattle, WA 98112 USA; 3 Smith-Root, Research Division, 16603 NE, 50th Avenue, Vancouver, WA 98686 USA; 4 Department of Biology, Western Washington University, Bellingham, WA 98225 USA; 5 Makah Fisheries Management, Neah Bay, WA 98357 USA; 6 Washington Department of Fish and Wildlife, Olympia, WA 98501 USA; 7 Fish Ecology Division, NOAA NMFS Northwest Fisheries Science Center, National Marine Fisheries Service, National Oceanic and Atmospheric Administration, 2725, Montlake Blvd. East, Seattle, WA 98117 USA; 8 Fishery Resource Analysis and Monitoring Division, NOAA NMFS Northwest Fisheries Science Center, National Marine Fisheries Service, National Oceanic and Atmospheric Administration, 2725, Montlake Blvd. East, Seattle, WA 98117 USA; 9 Department of Fisheries and Wildlife, Oregon State University, Corvallis, OR 97331 USA; 10 North Gulf Oceanic Society, 3430 Main St. Suite B1, Homer, Alaska 99603 USA

## Abstract

Many marine mammal predators, particularly pinnipeds, have increased in abundance in recent decades, generating new challenges for balancing human uses with recovery goals via ecosystem-based management. We used a spatio-temporal bioenergetics model of the Northeast Pacific Ocean to quantify how predation by three species of pinnipeds and killer whales (*Orcinus orca*) on Chinook salmon (*Oncorhynchus tshawytscha*) has changed since the 1970s along the west coast of North America, and compare these estimates to salmon fisheries. We find that from 1975 to 2015, biomass of Chinook salmon consumed by pinnipeds and killer whales increased from 6,100 to 15,200 metric tons (from 5 to 31.5 million individual salmon). Though there is variation across the regions in our model, overall, killer whales consume the largest biomass of Chinook salmon, but harbor seals (*Phoca vitulina*) consume the largest number of individuals. The decrease in adult Chinook salmon harvest from 1975–2015 was 16,400 to 9,600 metric tons. Thus, Chinook salmon removals (harvest + consumption) increased in the past 40 years despite catch reductions by fisheries, due to consumption by recovering pinnipeds and endangered killer whales. Long-term management strategies for Chinook salmon will need to consider potential conflicts between rebounding predators or endangered predators and prey.

## Introduction

Marine mammal population recoveries are a conservation success story in many parts of the world^1,2^. Multiple legal protections put in place in the mid-20^th^ century have resulted in recoveries of populations once threatened with extinction. For example, bans on whaling or prohibitions on imports of marine mammal products by the International Whaling Commission^3^, the Marine Mammal Protection Acts (US 1972, New Zealand 1978), Environmental Protection and Biodiversity Conservation Act (Australia 1999) and Species at Risk Act (Canada, 2002) have led to recoveries of some marine mammal populations. Further protections for some species were added under the US Endangered Species Act (ESA). In the Northeast Pacific Ocean, protection of marine mammals has led to recoveries of populations of humpback whales (*Megaptera novaeangliae*, 81 FR 62260; September 8 2016), and Steller sea lions (*Eumetopias jubatus*, 78 FR 66139; November 4 2013). Marine mammal populations never threatened with extinction also benefited from protection, with some populations recovering to high abundance levels (e.g. harbor seals *Phoca vitulina richardii*;^4,5^). Rates of recovery have been particularly strong for coastal species with relatively short generation times, such as pinnipeds: seals and sea lions^1^.

The unintended consequences of marine mammal recoveries have created new tradeoffs for natural resource managers to confront^6^. Examples of potential impacts of higher trophic level consumers on other species in the food web include: reduced recovery of forage fish such as Pacific herring (*Clupea pallasii*)^7^, increased competition between marine mammal species that share the same prey, such as pinnipeds and killer whales (*Orcinus orca*) in the Northeast Pacific^6^, and lastly, increased direct competition between marine mammal populations and fisheries. The potential impacts of recovering top predators on fisheries has been controversial. For example, within the International Whaling Commission (IWC), some argue that rebounding baleen whale populations are responsible for reductions in commercially fished prey populations and certain whale species should therefore be culled, whereas others argue that natural fluctuations in targeted fish populations and fisheries management are responsible for declines in yield^8^.

The recovery of pinnipeds in the coastal ecosystems of North America demonstrates all three of these potential conflicts. For example, populations of harbor seals and grey seals (*Halichoerus grypus*) on the coasts of North America have increased dramatically since the 1970s. Recent work using ecosystem models highlights the potential impacts that such recoveries may have on commercially fished species^9,10^. Like other generalist predators, quantifying the impact of these pinnipeds on prey species can be challenging because pinnipeds may consume fish at a variety of ages. For example, anadromous fish such as salmon may be consumed in estuaries as juveniles (as they leave streams to migrate to the ocean) or up to several years later as adults as they return to freshwater to spawn. A second challenge in quantifying the impact of these pinnipeds is that their diets vary in space and time, as predators alter their foraging to exploit local concentrations of prey.

In many other ecosystems around the world, there have been long-standing concerns about the potential impacts of marine predators on fisheries. On the west coast of the US and Canada, these concerns have been heightened because of external pressures on salmon populations (e.g. habitat loss). For example, over the last 20 years, multiple populations of Chinook salmon (*Oncorhynchus tshawytscha*), as well as Southern Resident and Northern Resident populations of salmon-eating killer whales, have been listed under the ESA or Canadian Species at Risk Act (SARA); the Southern Resident population is of particular concern due to both low population size and low population growth rate^11^. Studies examining conflicts between marine mammals and fisheries were initiated in the NE Pacific in the late 1970s after marine mammals caused losses in salmon fisheries^12^. Of the salmon species present on the west coast of North America, Chinook salmon are the largest and most valuable by weight. Chinook salmon migrate thousands of kilometers from their natal streams on the U.S. west coast to Alaska as juvenile fish, before returning 2–4 years later. The majority of salmon predation studies have focused on ‘hotspots’, including Puget Sound and the Columbia River, where there are apparent tradeoffs between local populations of pinnipeds and threatened or endangered salmon^13^. In most of these regions, genetic methods have recently been used to quantify the importance of salmon in diets of salmon-eating killer whales^14^ and pinnipeds^15^.

**Figure 1:**
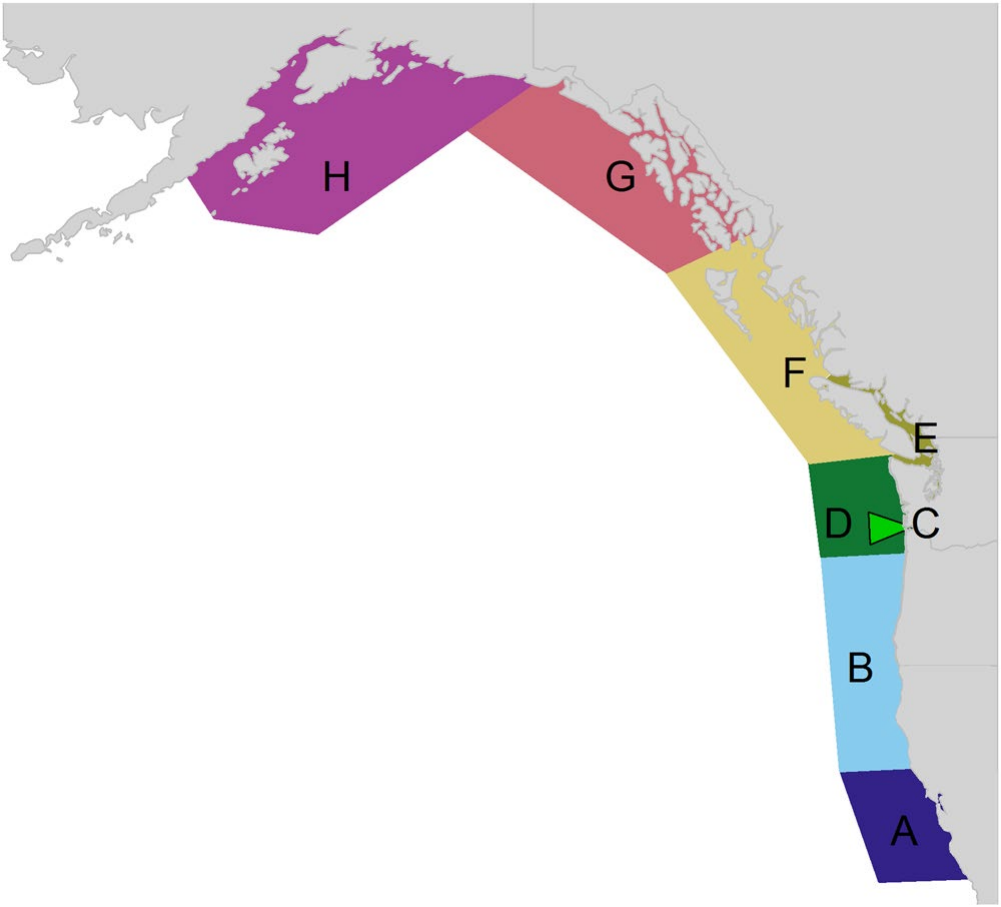
The eight areas in the study: Central California (**A**), northern California/Oregon (**B**), Columbia River (**C**), outer Washington coast (**D**), Salish Sea (**E**), West Coast of Vancouver Island and coastal British Columbia (**F**), Southeast Alaska (**G**), and the Gulf of Alaska (**H**). Map created using the maps package for R software^64^.

In the context of the global recovery of many marine mammals^1,2^, here we quantify how marine mammal predation on Chinook salmon has changed since the 1970 s along the west coast of North America (California to Alaska, including US and Canadian waters, Fig. 1), and compare this to salmon production and fishing mortality from commercial and recreational fisheries. Though Chinook salmon are consumed by a wide variety of predators, including birds, mammals, and other fish, the focus of our analysis is on the four marine mammal predators that have been previously documented to consume juvenile or adult Chinook salmon: harbor seals, fish-eating killer whales, California sea lions (*Zalophus californianus*), and Steller sea lions. Motivated in part by a recent peer-review of science to quantify the impact of salmon fisheries on Southern Resident killer whales^11^, and concerns about the timing and prey base required to recover such populations^16,17^, we place particular emphasis on interspecific competition between marine mammal species and specifically implications of changes for killer whales. We couple population data from the four marine mammal species and Chinook salmon to bioenergetics models and diet information. By examining spatial and temporal changes in consumption of Chinook salmon in the Northeast Pacific, we find evidence that salmon consumption by marine mammals has more than compensated for reductions in fisheries harvest from 1975–2015, which has implications for recovery of both endangered salmon and endangered killer whales.

## Results

**Figure 2:**
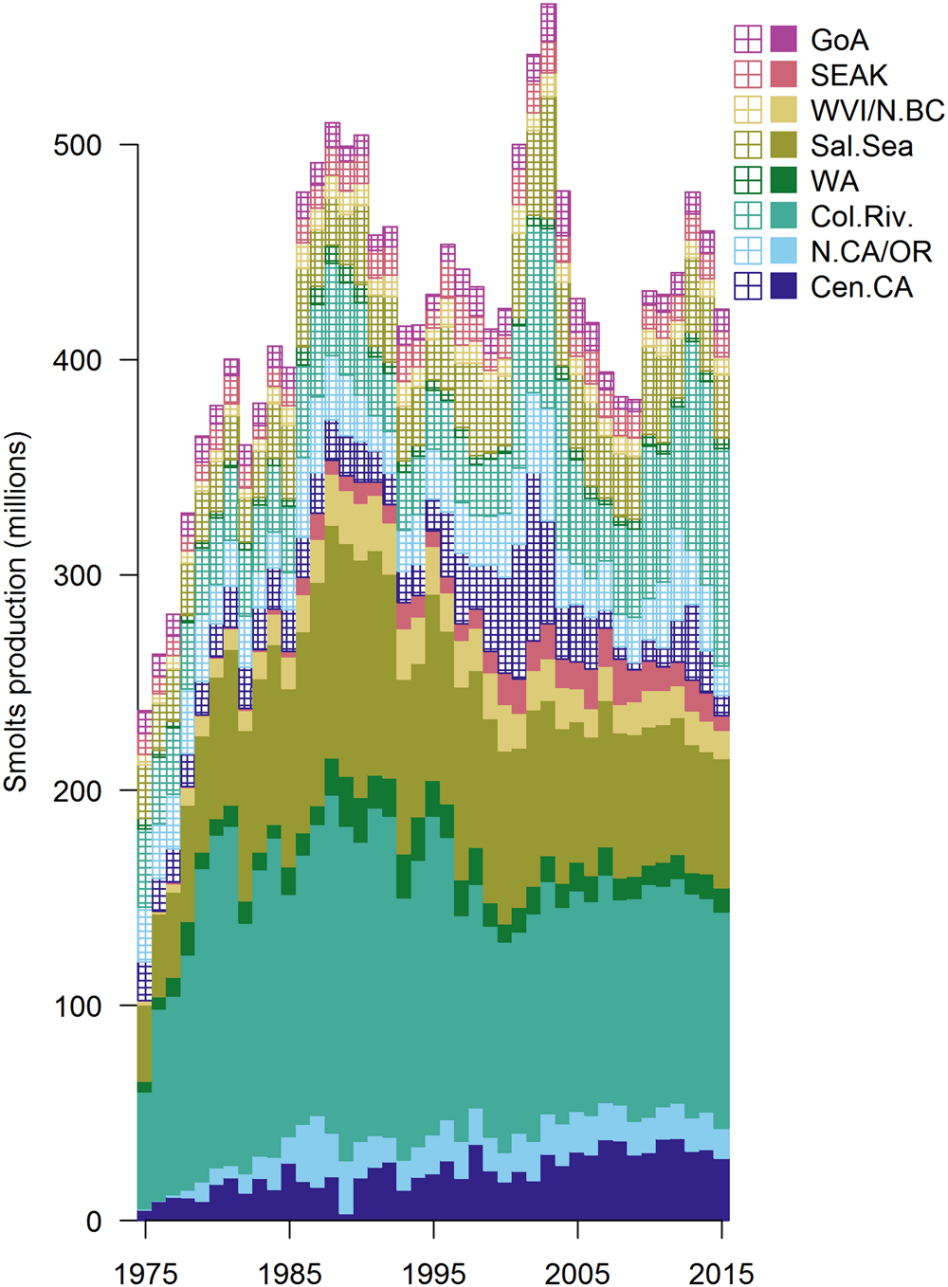
Natural (hatched) and hatchery (solid) Chinook salmon production by area between 1975 and 2015 for Central California (Cen.CA), Northern California/Oregon (N.CA/OR), Columbia River (Col. Riv.), outer Washington Coast (WA), Salish Sea (Sal. Sea), West Coast Vancouver Island and coastal British Columbia (WVI/N.BC), Southeast Alaska (SEAK), and Gulf of Alaska (GoA).

Total Chinook salmon smolt production on the west coast increased from the 1970s to the 1990s and has been relatively constant over the subsequent two decades (Fig. 2). Between 1975 and 2015 the estimated production of wild and hatchery Chinook salmon increased from 225 to 406 million juveniles (Fig. 2). In the 1970s and 1980s this was driven by an increase in production of hatchery fish. Since the mid 1980s, a decline in hatchery production has been offset by an increase in smolt production from some wild stocks, such as in the Columbia River.

**Figure 3:**
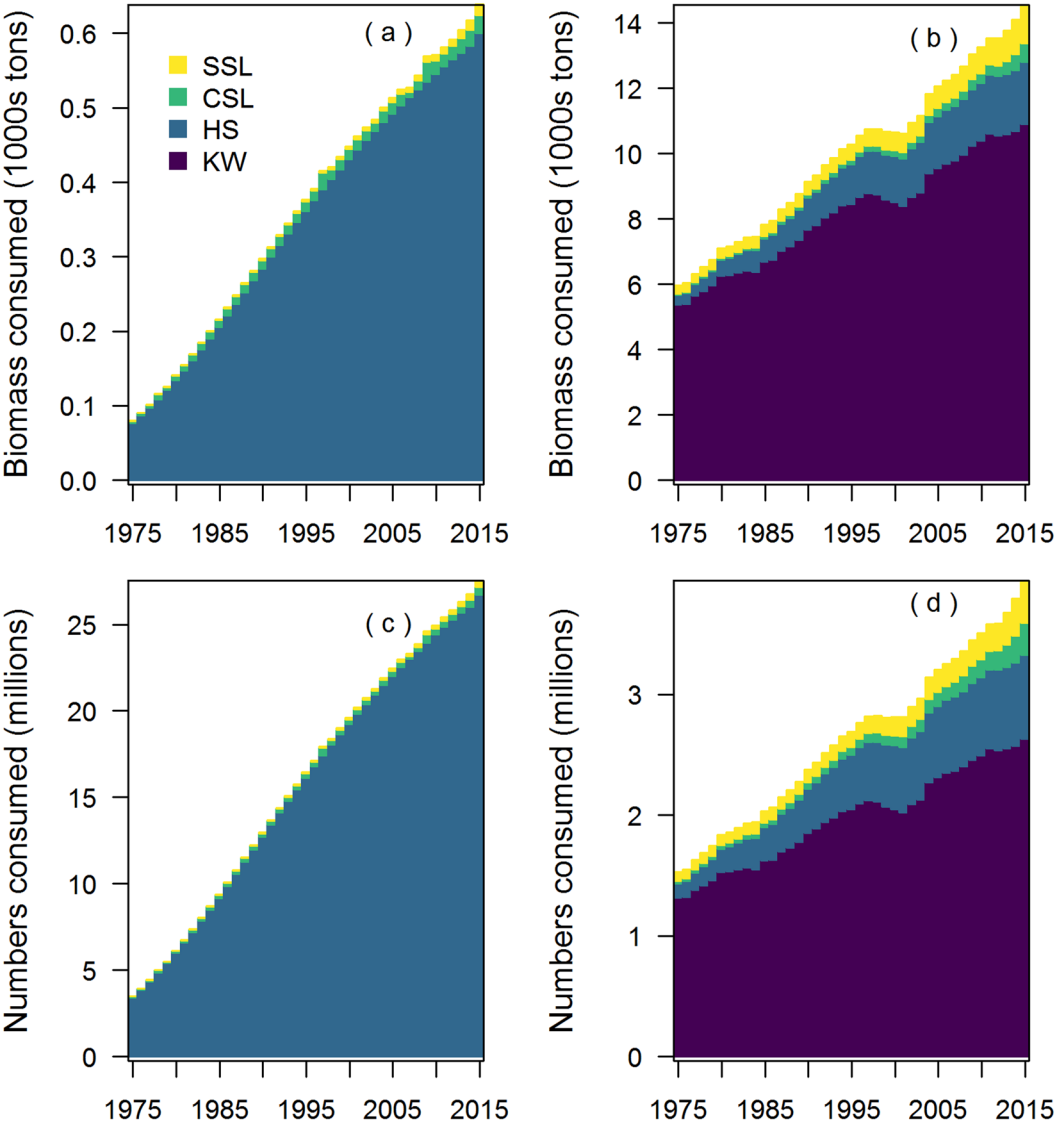
Consumption of Chinook salmon biomass ((**a**) juveniles, (**b**) adults ocean age one and greater) and total numbers ((**c**) juveniles, (**d**) adults ocean age one and greater) by killer whales (KW), harbor seals (HS), California sea lions (CSL), and Steller sea lions (SSL) from 1975 to 2015. Consumption is summed across all eight model areas shown in Fig. 1.

Chinook salmon biomass consumed by the marine mammal predators was estimated to have increased steadily over the entire study period from 6,100 to 15,200 metric tons (Fig. 3a,b). The estimated increase in predation was directly related to increasing predator abundance used in our model. Killer whales increased from 292 to 644 individual resident killer whales, harbor seals increased from 210,000 to 355,000, California sea lions increased from 5,900 to 47,000, and Steller sea lions increased from 74,400 to 78,500. Killer whales consumed the most Chinook salmon biomass (from 5,400 metric tons in 1975 to 10,900 metric tons in 2015), followed by harbor seals (400 to 2,500 metric tons), Steller sea lions (300 to 1,200 metric tons), and California sea lions (50 to 600 metric tons). Numerically, the predator consumption increased from 5 to 31.5 million individual Chinook salmon of varying ages (Fig. 3c,d). This was largely driven by increased consumption by harbor seals (from 3.5 million to 27.4 million individual Chinook salmon), followed by killer whales (1.3 to 2.6 million), California sea lions (0.1 to 0.7 million), and Steller sea lions (0.1 to 0.7 million).

Pinniped consumption of juvenile Chinook salmon was a substantial component of predation mortality coastwide, but particularly in the Salish Sea. Of the estimated 27.4 million Chinook salmon consumed coastwide by harbor seals in 2015 (Fig. 3), 23.2 million were smolts consumed in the Salish Sea. The percentage of the total coastwide smolt production consumed by harbor seals increased from 1.5% (3.5 million consumed out of 236.8 million estimated total production) in 1975 to 6.5% (27.4 million consumed out of 423.4 million estimated total production) in 2015. Harbor seals in the Salish Sea (i.e. Puget Sound, Strait of Georgia, and Strait of San Juan de Fuca) accounted for 86.4% of the total coast wide smolt consumption in 2015, due to large increases in the harbor seal abundance in this region between 1975 and 2015 (8,600 to 77,800), as well as a large diet fraction of Chinook salmon smolts relative to other regions (see supplemental material).

**Figure 4:**
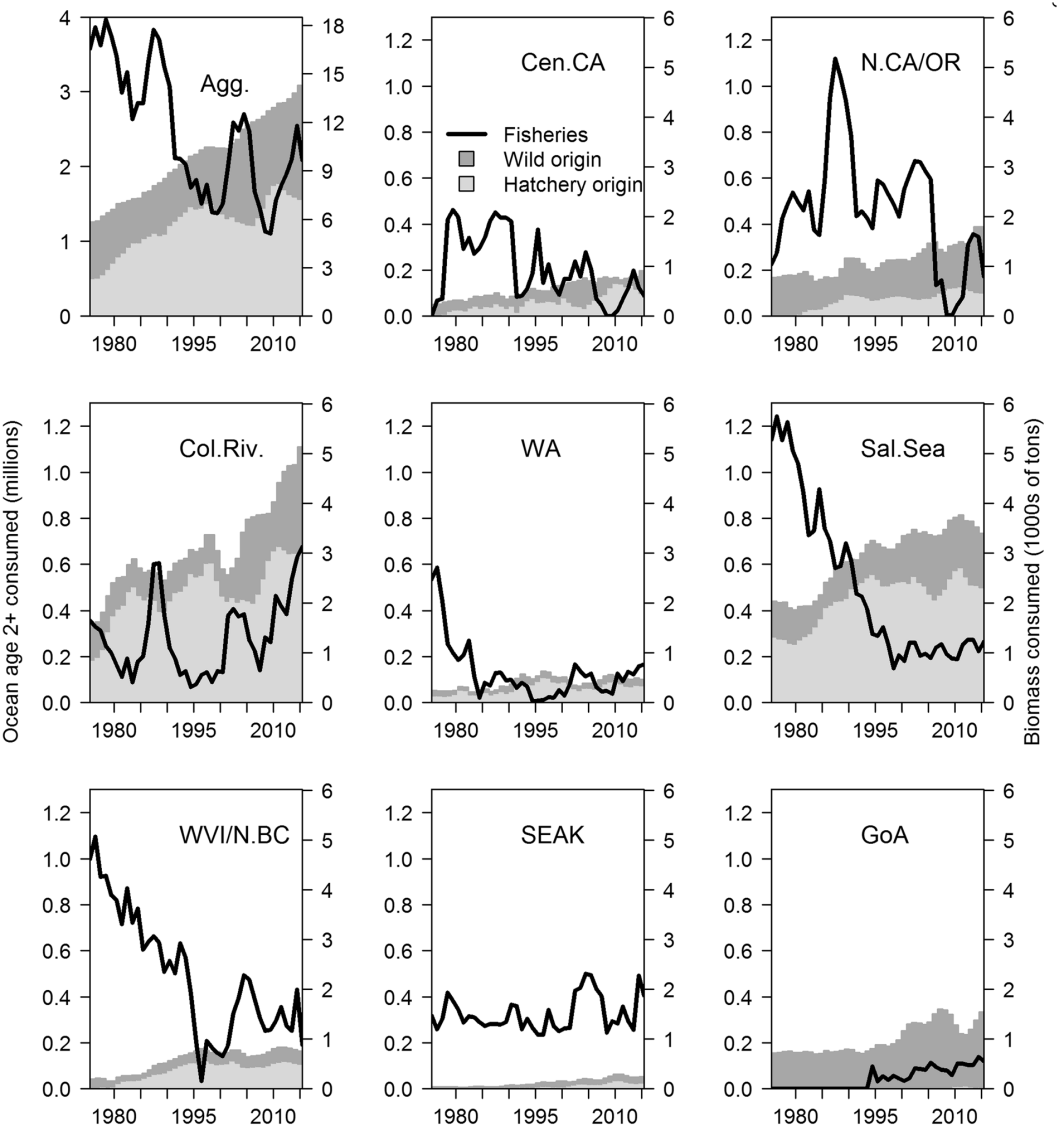
Total numbers (millions; primary axis) and biomass (thousands of metric tons; secondary axis) of adult Chinook salmon removed by fisheries (line) and the combined marine mammal predators (shaded areas) from 1975 to 2015. The top left panel sums over the whole model domain; each other panel represents hatchery and natural Chinook salmon stocks from a single area of origin. Note that estimates of predation in these panels include Chinook salmon consumed by marine mammals throughout the migratory range of that salmon stock (salmon originating in SEAK are consumed by marine mammals in Alaska, while salmon originating from Washington are potentially consumed by marine mammals in Washington, British Columbia, and Alaska).

While predation on Chinook salmon by marine mammal predators increased, annual harvest by commercial and recreational fisheries decreased from 3.6 million to 2.1 million individuals, equivalent to 16,400 to 9,600 metric tons (Fig. 4a). At the same time, predator consumption of Chinook salmon increased from 1.3 to 3.1 million adults (we exclude smolts and ocean age one jacks from the estimate because they are not retained in fisheries), or from 5,800 to 14,200 metric tons. The change in predation and harvest was not evenly distributed across Chinook salmon from different areas (Fig. 4). Generally, for Chinook salmon from natal stocks in the south (Central California, Northern California/Oregon, and Columbia River), predation impacts have increased strongly over time and exceeded harvest in recent years. These stocks’ longer migrations northward expose them to a gauntlet of predators throughout our modeled regions. Predation has also increased on Northern Chinook salmon stocks (Washington, W.Coast Vancouver Island and coastal British Columbia, and Southeast Alaska), but for these stocks predation is presently near or below the harvest. For Salish Sea Chinook salmon, strong increases in predation greatly exceed harvest; this is driven largely by local increases in pinniped abundance in the Salish Sea. Similarly, Chinook salmon from Gulf of Alaska stocks have experienced increasing predation (which exceeds harvest), due to local abundance of killer whales (including Gulf of Alaska and Southeast Alaska Resident killer whales).

**Figure 5:**
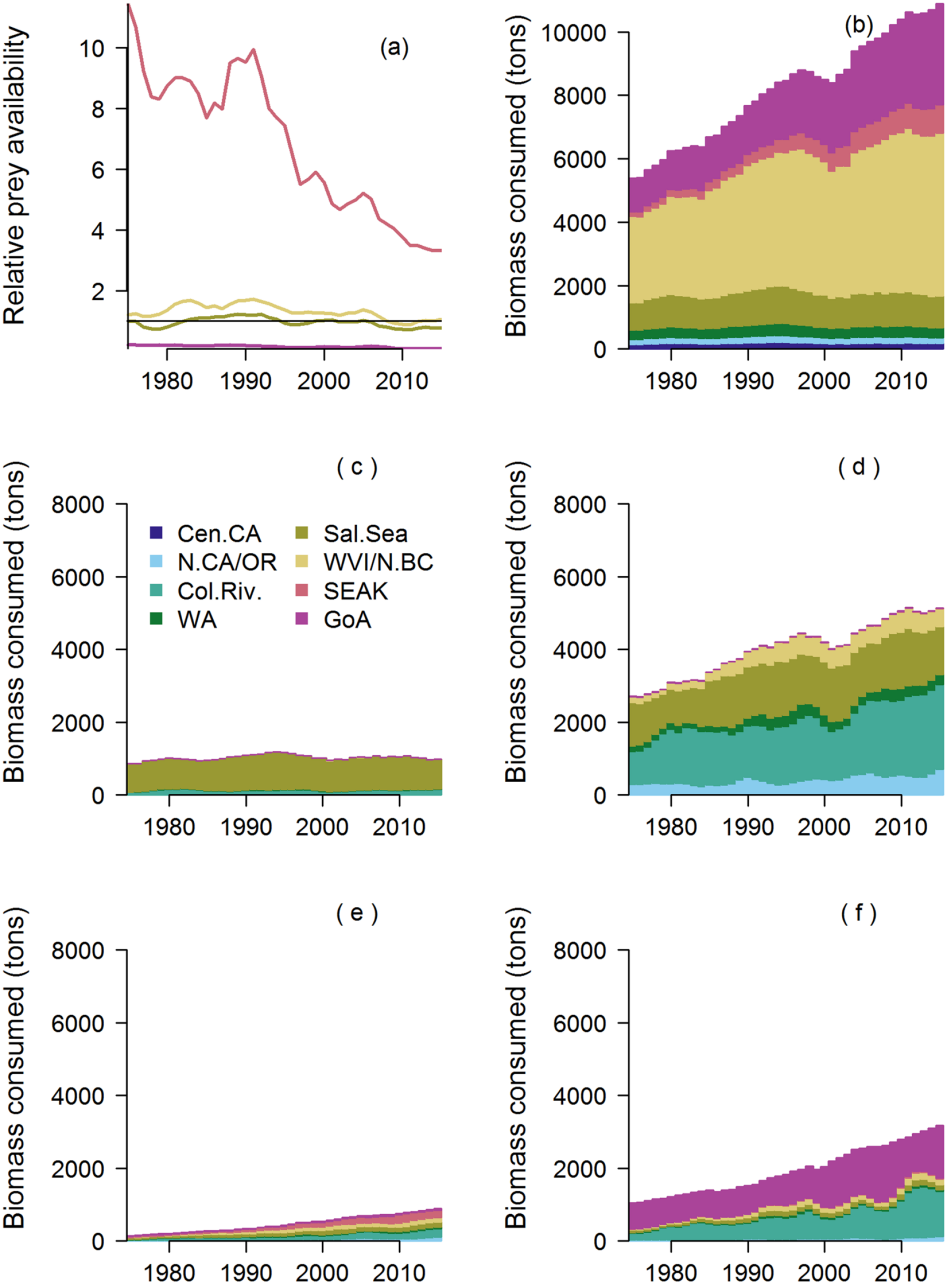
Estimates of the relative ratio of available adult Chinook salmon prey (equation (3)) to killer whale consumption of Chinook salmon in each ocean region ((**a**) equation (6)), estimated total Chinook salmon consumption by killer whales in each ocean region (**b**), and the estimated contribution of Chinook salmon originating from different ocean regions to composition of Chinook salmon consumed by killer whales in the Salish Sea (**c**), West Coast Vancouver Island and coastal British Columbia (**d**), Southeast Alaska (**e**), and Gulf of Alaska (**f**).

Killer whales are the largest consumers of Chinook salmon biomass among the predators in our model, accounting for 10,900 of the total 15,200 metric tons biomass consumed in 2015 (Fig. 5b). Since 1975, the largest increase in consumption has been from the Northern Resident killer whales along the West Coast Vancouver Island and coastal British Columbia (Fig. 5d), approximately 2,400 metric tons. The combined increase in consumption for the Routheast Alaska Residents and Western Alaska Residents from 1975 to 2015 was equal to about 2,900 metric tons. The Southern Resident population in the Salish Sea has remained relatively stable, and therefore the annual consumption within Salish Sea waters has been relatively constant at 900 to 1,200 metric tons, equivalent to about 190,000 to 260,000 adult Chinook salmon annually.

All regions exhibited declines in availability of Chinook salmon as prey for killer whales, even though killer whales in each region depend upon different Chinook salmon stocks. The ratio between Chinook salmon available as prey and the diet needs of the killer whales is estimated to have declined along the entire west coast during the last 40 years (Fig. 5a), although ratios for coastal British Columbia and Southeast Alaska were consistently higher than for the Salish Sea. We estimated that killer whales within each region depend upon Chinook salmon from distinct populations: the Southern Resident killer whale diets are dominated by Salish Sea Chinook salmon (Fig. 5c), Northern Resident killer whale diets are primarily Salish Sea and Columbia River Chinook salmon (Fig. 5d), Southeast Alaska Resident diets are more uniformly distributed across Chinook stocks from all regions (Fig. 5e), and Western Alaska Resident diets are likely to be dominated by Gulf of Alaska and Columbia River Chinook salmon stocks (Fig. 5f).

The Columbia River has previously been identified as an area with high marine mammal consumption of salmon^18^, and our results for this region illustrate the relative impacts of different predators and how this varies across salmon life stages. In 2015, harbor seals consumed just 14 metric tons of Chinook salmon versus the 219 and 227 metric tons consumed by California and Steller sea lions, respectively. Considering the consumption of just adult (ocean age two and older) Chinook salmon in 2015, we estimated that harbor seals consumed 1,000 adult Chinook salmon, California sea lions consumed 46,000, and Steller sea lions consumed 47,000. Harbor seals, however, likely prey substantially on out-migrating smolts, and we estimate they would have eaten 312,000 smolts in 2015.

**Figure 6:**
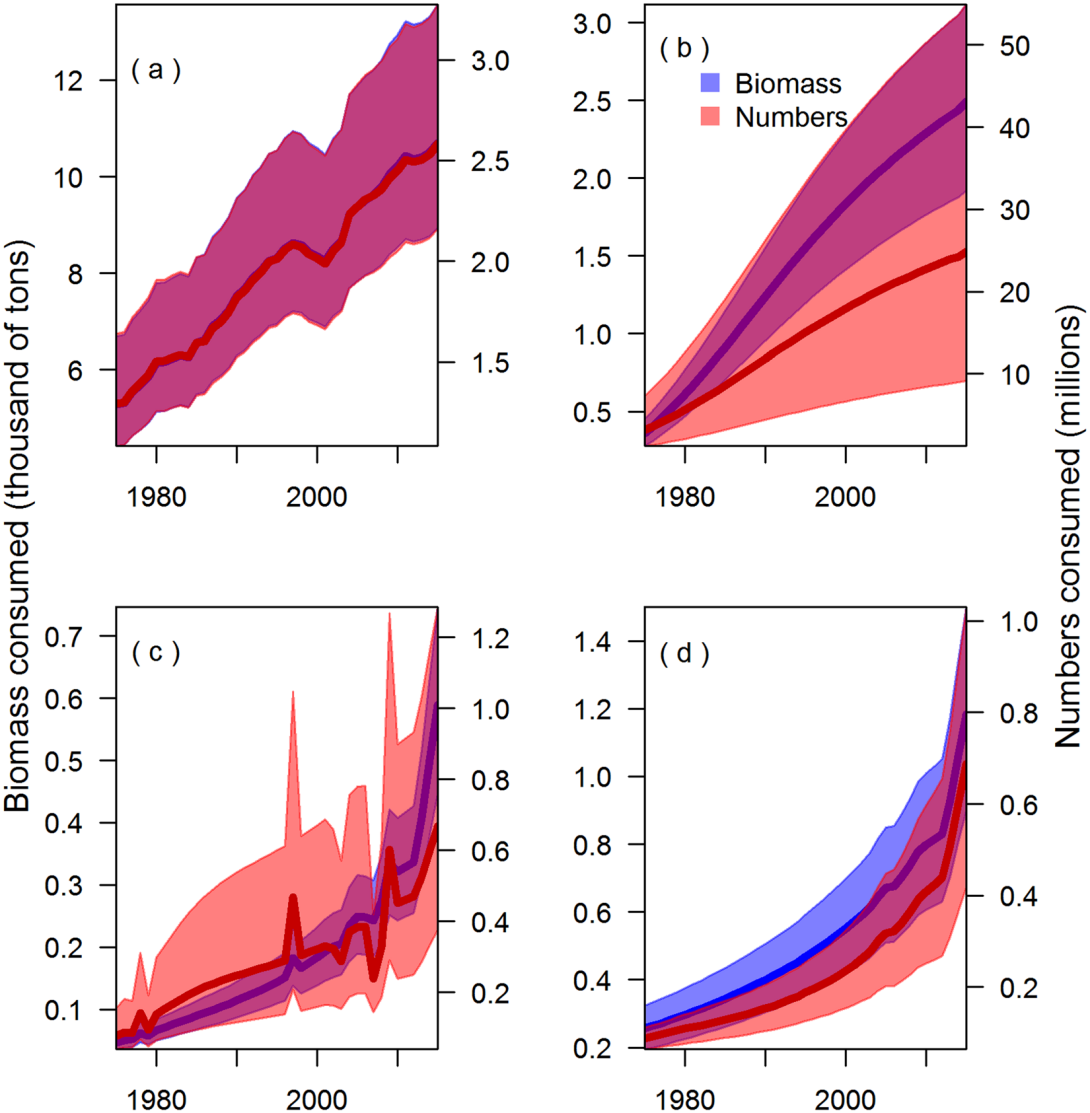
Estimates of consumption of Chinook salmon, with 95% confidence intervals, in terms of the annual biomass (primary axis; blue) and number (secondary axis; orange). Consumption by killer whales (**a**), harbor seals (**b**), California sea lions (**c**), and Steller sea lions (**d**). The red implies where the relative magnitude of uncertainty for the biomass and numbers overlap.

**Figure 7:**
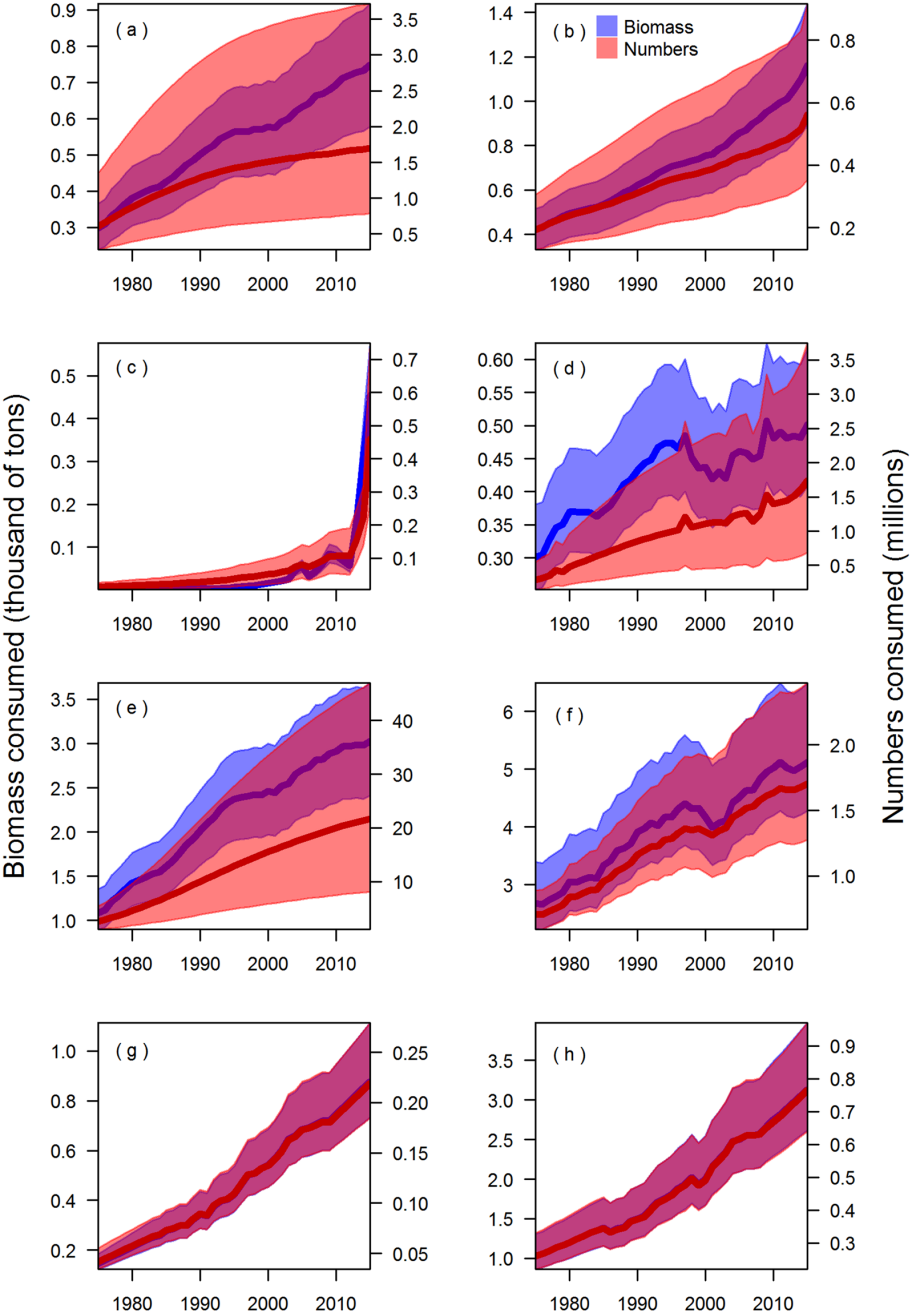
Estimates of consumption of Chinook salmon by the combined marine mammal predators, with uncertainty, in terms of the biomass (primary axis) and number (secondary axis) of Chinook salmon consumed per region: Central California (**a**), Northern California/coastal Oregon (**b**), Columbia River (**c**), Washington coast (**d**), Salish Sea (**e**), West Coast Vancouver Island and coastal British Columbia (**f**), Southeast Alaska (**g**), and Gulf of Alaska (**h**).

Including uncertainty in four key parameters related to predator abundance, diets, and bioenergetics does not qualitatively change the trends and relative impacts of the predators described above. Given uncertainty in these parameters, the estimated total biomass of Chinook salmon consumed in 2015 was between 12,400 and 18,700 metric tons for 95% of the simulations. The total number consumed varied between 12.5 million and 59.8 million individuals; this has higher relative uncertainty than biomass because it additionally incorporates uncertainty in smolt size and smolt fraction parameters. In 2015, approximately half of the uncertainty in the estimated total biomass of Chinook salmon consumed by marine predators can be attributed to killer whales (8,900 to 13,600 metric tons, Fig. 6a), while almost all of the uncertainty in the total number of Chinook salmon consumed can be attributed to harbor seals (9.2 to 54.9 million individuals, Fig. 6b). Across areas there is a similar pattern of uncertainty related to these predators (Fig. 7): in 2015 coastal British Columbia had the largest killer whale population among areas (261) and it also had the largest uncertainty in biomass consumed (4,300 to 6,500 metric tons; Fig. 7f), while the Salish Sea had the largest harbor seal population (78,000) and largest uncertainty in the number of Chinook salmon consumed (8.1 to 46.9 million) (Fig. 7e).

## Discussion

Competition between fisheries and predators, such as marine mammals, has been a concern around the world, particularly as recent increases in predator populations have coincided with declines in some of their fish prey populations. Most studies attempting to quantify fishery losses to predation or damage to fishing gear caused by marine mammals have been localized to hotspot areas of conflicts. Our spatio-temporal model of marine mammal – Chinook salmon interactions is novel in that we quantified consumption of a highly migratory fish species by marine mammals over a broad spatial range, and across the entire fish life cycle in marine and estuary waters. We estimate that marine mammal consumption of Chinook salmon has increased dramatically over the past 40 years, and may now exceed the combined harvest by commercial and recreational fisheries in Central California, Northern California/Oregon, the Columbia River, the Salish Sea, and the Gulf of Alaska (Fig. 4).

Our main finding, that marine mammal consumption of Chinook salmon has increased dramatically over the last 40 years and likely exceeds removals by fisheries, was robust to a range of uncertainties in input parameters. Link *et al*.^19^ identify the need and challenge of addressing uncertainty in ecological models; in the framework of those authors we primarily addressed parameter uncertainty stemming from observation error and natural variability in key aspects of Chinook salmon and marine mammal biology. Though we did not address structural uncertainty in the model formulation, for instance by applying a multi-model framework^19–21^, this would be possible by comparing our bioenergetics approach to other methods such as individual-based models^22^ or time series modeling approaches^23^. Best practices for applications of ecological models^24^ suggest consideration of multiple models, addressing parameter uncertainty, and understanding that models such as ours are strategic tools to identify major tradeoffs and explore hypotheses.

### Implications for salmon recovery

Increased consumption demand of growing marine mammal populations in the Northeast Pacific could be masking the success of coastwide salmon recovery efforts. For example, long term reductions in the salmon available for commercial and recreational fisheries may not reflect lower abundance of salmon, but rather a reallocation from human harvest to marine mammal consumption. Because many populations of Chinook salmon in the Northeast Pacific are of conservation concern, substantial resources have been invested to improve salmon passage through hydropower dams^25^, restore salmon habitat^26^, reduce fishing^27^, and otherwise improve conditions in rivers and streams to improve productivity. Collectively, these recovery efforts may have increased Chinook salmon survival or recovery, but these increases in salmon populations may be offset by salmon consumption by more-rapidly increasing populations of marine mammals and other predators. Samhouri *et al*.^17^ point out the challenges of this type of “predator-first” recovery, versus synchronous recovery of both predator and prey.

Predation of Chinook salmon by marine mammals in well-studied ‘hotspots’ (e.g. Salish Sea, Columbia River) is well known, but our results suggest additional predation in the ocean may be greater than previously documented. For instance, our estimates of in-river consumption of adult (ocean age two and greater) Chinook salmon by sea lions in the Columbia River from January to August in 2015 is 65,000 (49,000–81,000), which is lower than the most recent direct, tagging-based estimate of 95,000 (61,000–127,000) spring/summer Chinook (Michelle Rub, pers. comm, August 7^th^, 2017). However, our model estimates sea lions consumed approximately 70,000 ocean age one Chinook salmon during the same period. Chinook salmon are also highly migratory, and our model allows salmon to be susceptible to predation throughout their range. Each salmon population has a unique distribution in the ocean^28^, which affects the encounter rates by fisheries and predators; for example, southern Chinook salmon populations in our model have longer migrations and generally were susceptible to a larger number of marine mammal populations and associated predation. From the perspective of predator populations or fisheries, northern regions have a wider portfolio of Chinook salmon populations available to harvest^29^.

Though not a direct focus of our analysis, approximately half of the Chinook salmon consumed by marine mammals or available to fisheries are of hatchery origin. Hatchery releases are conducted to increase fishing opportunities and assist salmon recovery efforts by helping supplement wild populations of conservation concern^30^. An unintended effect of these programs is that they may contribute to unforeseen interactions between wild and hatchery origin fish. Though our model did not include different predation rates depending on salmon origin (hatchery, wild), it is possible that hatchery origin salmon provide a subsidy for marine mammals, leading to a numerical response in these predators and ultimately an increase in predation rates on wild fish. Alternatively, if marine mammals are generalist predators that lack a strong numerical response to increased salmon abundance, but nonetheless prefer Chinook salmon and seek them out relative to other prey, then hatchery Chinook salmon could lessen predation rates on wild fish. Exploring these dynamics would require modeling the functional response between Chinook salmon and marine mammal predators, and may be a fruitful avenue for future research.

### Implications for killer whale recovery

The abundance and diversity of Chinook salmon populations available as prey may have particular significance to predator populations that specialize on these populations as prey, such as fish-eating killer whales. Multiple populations of killer whales occur throughout the migratory range of Chinook salmon. Most of the salmon populations originating from natal streams on the west coast of the US and Canada migrate northward to Alaska, so killer whale populations inhabiting Alaskan waters have a much broader range of salmon populations available as prey. In contrast, the most southern population of killer whales (Southern Resident killer whales), distributed in the Salish Sea and west coast of the US, is the most at-risk population with a long-term growth rate close to zero^11^, and a much smaller diversity of salmon populations available as prey. This narrower selection of Chinook salmon stocks available to Southern Resident killer whales may be a competitive disadvantage compared to higher latitude killer whale populations. Increasing consumption of Chinook salmon by pinnipeds may also be limiting the growth of the Southern Resident killer whale population. Our results suggest that at least in recent years competition with other marine mammals is a more important factor limiting the growth of this endangered population than competition with human fisheries.

### Future work

Though we conducted full sensitivity analyses on important model parameters, there are some potential sources of error that were not included. One obvious uncertainty is the ocean distribution of Chinook salmon. Available data on Chinook salmon distribution (following^28^) may not fully describe the temporal and spatial overlap between predators and Chinook salmon (particularly juveniles), especially during the period of rapid growth during the first months in salt water^31^. While higher resolution of the temporal and spatial distribution of salmon populations would be useful, the geographic range and high rates of mortality make tracking fine scale movement and distributions difficult. Currently the best available information is based on coded wire tags recovered from commercial and recreational fisheries, not a systematic sample of the Chinook salmon distribution. Further, these distributions are assumed constant across years, which may be unrealistic, particularly if Chinook salmon have experienced distribution shifts in response to recent fluctuations in marine conditions^32^. A second potential source of error is in the proportion of Chinook salmon in predator diets. Data on diet fraction are informed by recent syntheses^9,33^ and updated field and laboratory methods^14,15^, but nonetheless future work could consider more ecologically realistic (but complex) functional responses that include flexible diets of predators. For generalist predators such as pinnipeds, this would necessitate modeling multiple prey species. A third important assumption is that individual populations of Chinook salmon have not experienced trends in mean length, weight, or energetic content. Long term studies of Chinook salmon sizes in the ocean have shown significant reductions in growth rates (length-at-age^34^ and weight-at-age^35,36^ of adult Chinook salmon with the exact mechanism for this decline not known. Because the relationship between fish length and weight (or energy) is non-linear, small decreases in adult length can lead to large differences in the number of salmon consumed – this is particularly true for killer whales^37,38^.

Bioenergetics models such as ours are dependent on both historic and contemporary data collection efforts. This is particularly true for our spatially and temporally explicit model, because many parameters vary seasonally or over geographic regions. In using models such as ours to provide guidance to decision makers about tradeoffs, it is important for estimates to be both accurate and precise. Though it may be unrealistic to collect data on predators and prey at a fine scale, overall uncertainties would be reduced by balancing samples between historic predation hotspots and regions that have lower densities of predators but nonetheless substantial predation rates (e.g. coastal British Columbia, pinnipeds in the lower Columbia River). This will improve predictions about future impacts of predation on salmon and salmon fishing, and about tradeoffs and conflicting objectives of mandates such as the Marine Mammal Protection Act and the Endangered Species Act. Furthermore, our bioenergetics model does not “close the loop” on the full life-cycle of the Chinook salmon. Such an effort may provide estimates of the competitive interactions between the marine mammals and fishing sectors - instead of the relative ranking of their demands which we have provided. However, this future effort would require a more detailed reconciliation of the differences between the temporal and spatial availability of Chinook salmon to the marine mammals and fisheries, and more detailed estimates of the escapement and smolt production for the wild stocks to create a feedback between consumption and future production.

## Methods

### Model overview

We estimated the consumption of Chinook salmon by killer whales, harbor seals, and California and Steller sea lions to determine the location, source, and timing of predation mortality in the eastern Pacific, 1975–2015. Using bioenergetics models and information regarding marine mammal diets, we calculated this predation demand, and we then transformed the amount of energy each predator derived from Chinook salmon into estimates of biomass and numbers of Chinook consumed. Because marine mammal predators consume Chinook salmon of different sizes/ages, we used a Chinook salmon life cycle model to link the cohort abundance to predation demands in space and time. We provide a detailed description of the data in the appendix, and reserve the methods for describing how the data are incorporated into the bioenergetics model.

### Predator dynamics, distribution, and energy demands

Estimates of marine mammal abundance were based on surveys by state and federal agencies (see supplemental material of model input). Years with missing survey data were interpolated by fitting logistic or exponential models to the survey data. Killer whales and sea lions are highly mobile, including migrations beyond their core ranges, and we therefore assembled information in the literature to determine temporal/spatial distribution of these species across areas and over seasons. Examples of detailed temporal/spatial patterns include that Southern Resident killer whales (SRKW) feed in the Salish Sea during the summer but leave during the winter months^39^, Northern Resident killer whales occupy the waters of west Vancouver Island and British Columbia coast^40,41^, and Southeast Alaska Residents and Gulf of Alaska Residents split their time in different areas of Alaska^42^. California and Steller sea lion populations in the Salish Sea and Columbia River areas exhibit a bi-modal distribution – feeding on spring and fall runs of returning adult salmon and returning to colonies along the outer coasts in summer and winter. Harbor seals in each region were assumed to be resident^43^ with no exchange between populations in adjacent areas.

Because a predator’s energy demand is determined by its mass^44^, we combined weight-at-age models with information about the abundance, sex and age structure of the population to determine the total mass of the predator population in each area. For Southern Resident killer whales the age and sex distributions are known with perfect detection^45^, but populations in Northern British Columbia and Southeastern and the Central Gulf of Alaska^42^ are estimated based on mark-recapture observation with imperfect detection. Sex and age distributions for harbor seals^46^, California sea lions^47^ and Steller sea lions^48^ were estimated from survival tables; however, only California sea lions age six and older are assumed to consume Chinook salmon (pers. comm. J. Laake, NOAA AFSC). In some regions, such as the Salish Sea^49^ or Columbia River, populations of sea lions are thought to be predominantly male, and thus females were excluded from these model regions.

Chasco *et al*.^9^ developed a modeling framework that calculated bioenergetics (energy) needs for the four marine mammals in Puget Sound. Here we apply an extended version of that model for the eight regions in the broader northeast Pacific. Daily energy (${\rm{E}}{{\rm{D}}}_{p,h,j,y,t,i,a,s}$; Eq. 1; kcal/day) obtained through consumption of Chinook salmon for a predator population was estimated using Kleiber’s model of basal metabolic rate^44^. To transform BMR into field metabolic rate (FMR), which accounts for species and sex specific activity patterns, we multiplied the Kleiber model by a dimensionless scaling factor (${\alpha }_{p,i,s}$) for killer whales^50^, harbor seals^51^, California sea lions^52^ and Steller sea lions^48^. Total needs were calculated by multiplying these factors by the product of the predator abundance (${{\rm{N}}}_{p,h,y}$), the proportion of a predator population that is age *i* (${\rm{P}}{{\rm{A}}}_{p,h,y,i}$), sex ratio (${\mathrm{PF}}_{p,h,y,i}$), fraction of total energy derived from Chinook salmon (${\mathrm{FEC}}_{p,j,t}$), the selectivity of different age Chinook salmon (${\mathrm{SEL}}_{p,j,t,a}$), and the matrix describing the temporal and spatial distribution (${{\rm{\Phi }}}_{p,h,j,t,s}$),

1 $${\bf{E}}{{\bf{D}}}_{{\boldsymbol{p}},{\boldsymbol{h}},{\boldsymbol{j}},{\boldsymbol{y}},{\boldsymbol{t}},{\boldsymbol{i}},{\boldsymbol{a}},{\boldsymbol{s}}}={{\boldsymbol{\Phi }}}_{{\boldsymbol{p}},{\boldsymbol{h}},{\boldsymbol{j}},{\boldsymbol{t}},{\boldsymbol{s}}}\times {\bf{S}}{\bf{E}}{{\bf{L}}}_{{\boldsymbol{p}},{\boldsymbol{j}},{\boldsymbol{t}},{\boldsymbol{a}}}\times {\bf{F}}{\bf{E}}{{\bf{C}}}_{{\boldsymbol{p}},{\boldsymbol{j}},{\boldsymbol{t}}}\times {{\bf{N}}}_{{\boldsymbol{p}},{\boldsymbol{h}},{\boldsymbol{y}}}\times {\bf{P}}{{\bf{A}}}_{{\boldsymbol{p}},{\boldsymbol{h}},{\boldsymbol{y}},{\boldsymbol{i}}}\times {\bf{P}}{{\bf{F}}}_{{\boldsymbol{p}},{\boldsymbol{h}},{\boldsymbol{y}},{\boldsymbol{i}}}\times \frac{{{\boldsymbol{\alpha }}}_{{\boldsymbol{p}},{\boldsymbol{i}},{\boldsymbol{s}}}{{\bf{M}}}_{{\boldsymbol{p}},{\boldsymbol{h}},{\boldsymbol{i}},{\boldsymbol{s}}}^{0.75}}{{\bf{E}}{{\bf{f}}}_{{\boldsymbol{p}}}}$$

Where the subscripts for the model are: predator *p*, predator age *i*, sex *s*, that originated from area *h*, and occupies location *j* during year *y* time-step *t*, and prey on Chinook salmon of age *a*. The mass-at-age (*M*_*p,h,i,s*_; kg) for killer whales^50^, harbor seals^53^, and California sea lions^54^ and Steller sea lions^55^ are all based on published estimates in the literature. The bioenergetics constant in the power function is assumed to be 0.75 for all predators^56^. We used an average digestive efficiency (Ef_p_) of 0.875^48,51^ for California and Steller sea lions, 0.825 for harbor seals^51^, and 0.847 for killer whales^50^. The model also assumes that the predators consumed the entire Chinook salmon, regardless of size.

Selectivity (SEL_*p,j,t,a*_) and fraction of energy comprised of Chinook salmon (FEC_*p,j,t*_) for predators were based on a search of over 300 peer-reviewed journals and scientific reports^33^, and updated in 2017 with additional publications and technical reports as indicated in the Appendix. Selectivity describes the size classes of salmon consumed by each predator species, and is relevant because a juvenile Chinook salmon has about three orders of magnitude less energy than the average adult Chinook salmon^9^. Fraction of energy comprised of Chinook salmon (FEC_*p,j,t*_) would ideally be based on diet composition by percent energy or percent weight. However, the majority of the diet composition information for pinnipeds in the literature is based instead on frequency of occurrence (FO) observations, which is problematic because FO data do not sum to one, and many studies reporting FO do not partition salmon to species level. To transform pinniped FO into split sample frequency of occurrence (SSFO), a more useful proxy because the diet fractions sum to one, we used paired observations between FO and SSFO in Thomas *et al*.^15^. To disaggregate observations of total salmon consumed into Chinook salmon and other salmonids, we assumed the ratio of Chinook salmon to other salmonids from available species-specific harvest data in each area. This is a reasonable assumption since pinnipeds are considered to be generalist predators that are likely to select salmon species in proportion to the numbers present.

### Salmon production, timing and distribution

Our model uses a monthly time-step *t* to track the number of Chinook salmon of age *a* in location *j* that originated from area *h* based on the attributes of run type *r* and origin *o*. The production of the Chinook salmon for a particular cohort (S_*h,j,r,o,m,y,t,a* = 1_) in the model is based on the annual smolt production (R_*h,r,o,y*_) reported in the Regional Mark Information System database^57^ for hatchery fish, and spawner escapement estimates from agency reports^27^ for naturally spawning fish. Natural smolt production was estimated to be the spawner abundance, divided by two to yield female spawners, then multiplied by the average number of smolts produced-per-female Chinook salmon. There are very limited data on the smolts produced-per-female, and they are highly variable both within and between area tributaries (see review in supplemental material): we assumed an average of 220 smolts produced per female across all years and areas. The timing of juvenile Chinook salmon emigration from freshwater to the marine environment was based on hatchery release coded-wire-tag (CWT) information in the RMIS database. We assumed that hatchery and natural origin fish had the same migration timing for a given run type. We also assumed that the average lag between release date and their arrival in the near-shore areas was less than a month: that is, the month that a juvenile was released was the month that it entered the ocean.

The size of the juvenile Chinook salmon is important in estimating the number of juveniles consumed. Not only do juveniles grow during their down river migration, but they also grow during the occupancy in each area (areas A-H, Fig. 1) which can last for several months^58^. Although our model consists of monthly time-steps related to predator consumption, tracking monthly cohorts of juvenile salmon from each of the tributaries along the eastern Pacific is beyond the scope of this analysis. We assumed that the average juvenile spends 10 days migrating down river and an additional one month (30 days) in each area. To account for this period of growth we assumed the average juvenile grows 1.0 mm/day^59^, thus adding an additional 40 mm of length to the average juvenile release size. To account for variability in juvenile size, we assume the juvenile lengths are log-normally distributed with a standard deviation of 0.5 in our sensitivity analysis.

### Predator-prey dynamics

The combination of predator and prey movement, as well as both natural and predation mortality, make the order of operations within a time-step important. From the Chinook salmon’s perspective, the order in each time-step is as follows: 1) Chinook salmon distribute themselves across the areas based on the spatial transition matrix, followed by 2) natural mortality, 3) predation morality, and finally 4) escapement. The number of Chinook salmon at the beginning of each time-step ($S$) is the equal to the total abundance of wild and hatchery salmon at the end of the previous time step ($S^{\prime\prime}$), times the fraction of Chinook salmon from area *h* that are distributed to location *j* (${{\rm{\Theta }}}_{h,j,r}$),

2 $$\begin{array}{llll}{{\rm{S}}}_{h,j,r,o,m,y,t,a} & = & {{\rm{\Theta }}}_{h,j,r}\,{\sum }_{j}{{\rm{S}}}_{h,j,r,o,m,y,t-1,a}^{^{\prime\prime} }, & t > 1,a > 0\\  & = & {{\rm{MAT}}}_{h,r,o,m,a}\,{\sum }_{j}{{\rm{S}}}_{h,j,r,o,m,y-1,nt,a-1}^{^{\prime\prime} }, & t=1,a > 0\end{array}$$

The spatial transition matrix, ${{\rm{\Theta }}}_{h,j,r}$, is based on Weitkamp^28^ and describes the recovery location for tagged hatchery Chinook salmon in commercial and recreational fisheries throughout the eastern Pacific. The migratory state (*m*) of a Chinook salmon in a particular age class is determined at the beginning of the year (*t* = 1) and describes the conditional probability of a Chinook salmon maturing at age *a* (MAT_*h,r,a*_) based on the Fishery Regulation Assessment Model (FRAM;^60^). When a Chinook salmon changes from an immature to a mature state, it remains in that state throughout the year. Escapement refers to the mature salmon that leave the ocean pool to return to spawn in the natal tributaries. The number at the end of the time-step is equal to the number of salmon after predation ($S^{\prime}$) minus the escapement,

3 $$S{\text{'}\text{'}}_{h,j,r,o,m=2,y,t,a}={S\text{'}}_{h,j,r,o,m=2,y,t,a}(1-{{\rm{ESCT}}}_{j,r,t}),\,t < nt$$

where, ESCT_*j,r,t*_ is the fraction of the mature (m = 2) population leaving marine waters in that time-step. The average escapement timing was based on the summaries of west coast Chinook salmon populations^61^: escapement timing was assumed to vary by run type, but not area or year.

The number of Chinook salmon remaining after predation is equal to number at the beginning of the time-step times natural survival, which accounts for factors such as disease and consumption by predators not explicitly considered here, (*surv*_*t,a*_), minus the number consumed by predators (NC_*p,h,j,r,o,m,y,t,a*_),

4 $${S\text{'}}_{h,j,r,o,m,y,t,a}=sur{v}_{t,a}{{\rm{S}}}_{h,j,r,o,m,y,t,a}-\,{\rm{\min }}({\sum }_{p}{{\rm{NC}}}_{p,h,j,r,o,m,y,t,a},\,0.95\,sur{v}_{t,a}{{\rm{S}}}_{h,j,r,o,m,y,t,a})$$

The proportion surviving, *surv*_*t,a*_, was assumed to vary by age and time-step^60^, but not by run, origin, year, or migratory state. To avoid instances where the total consumption across all predators may exceed the available numbers of Chinook salmon, we assumed that the maximum consumption rate was 95%. The predation mortality is defined as,

5 $${\bf{N}}{{\bf{C}}}_{{\boldsymbol{p}},{\boldsymbol{h}},{\boldsymbol{j}},{\boldsymbol{r}},{\boldsymbol{o}},{\boldsymbol{m}},{\boldsymbol{y}},{\boldsymbol{t}},{\boldsymbol{a}}}=\frac{{\sum }_{{\boldsymbol{s}}}{\sum }_{{\boldsymbol{i}}}{\bf{E}}{{\bf{D}}}_{{\boldsymbol{p}},{\boldsymbol{h}},{\boldsymbol{j}},{\boldsymbol{y}},{\boldsymbol{t}},{\boldsymbol{s}},{\boldsymbol{i}},{\boldsymbol{a}}}}{{\bf{E}}{{\bf{C}}}_{{\boldsymbol{h}},{\boldsymbol{r}},{\boldsymbol{o}},{\boldsymbol{t}},{\boldsymbol{a}}}}{\bf{F}}{{\bf{S}}}_{{\boldsymbol{p}},{\boldsymbol{h}},{\boldsymbol{j}},{\boldsymbol{r}},{\boldsymbol{o}},{\boldsymbol{y}},{\boldsymbol{t}},{\boldsymbol{m}},{\boldsymbol{a}}}$$

where, the number of Chinook salmon consumed (NC_*p,h,j,r,o,m,y,t,a*_; d^−1^) was based on: 1) the amount of energy derived from Chinook salmon (ED_*p,h,j,y,t,s,i,a*_; kcal d^−1^), 2) the energetic content of an individual Chinook salmon from a particular cohort (EC_*h,r,o,t,a*_;^62^; kcal) which is a function of length-at-age throughout the year^60^, and 3) the relative abundance of Chinook salmon cohorts in area *j* during time-step *t* (FS_*p,h,j,r,o,y,t,m,a*_). Energetic content of Chinook salmon^62^ varies by length but not by sex. Relative abundance of Chinook salmon cohorts in area *j* during time-step *t* is computed as:

6 $${\bf{F}}{{\bf{S}}}_{{\boldsymbol{h}},{\boldsymbol{j}},{\boldsymbol{r}},{\boldsymbol{o}},{\boldsymbol{y}},{\boldsymbol{t}},{\boldsymbol{m}},{\boldsymbol{a}}}=\frac{{{\bf{S}}}_{{\boldsymbol{h}},{\boldsymbol{j}},{\boldsymbol{r}},{\boldsymbol{o}},{\boldsymbol{y}},{\boldsymbol{t}},{\boldsymbol{m}},{\boldsymbol{a}}}}{{\sum }_{{\boldsymbol{h}}}{\sum }_{{\boldsymbol{r}}}{\sum }_{{\boldsymbol{o}}}{\sum }_{{\boldsymbol{y}}}{\sum }_{{\boldsymbol{t}}}{\sum }_{{\boldsymbol{m}}}{{\bf{S}}}_{{\boldsymbol{h}},{\boldsymbol{j}},{\boldsymbol{r}},{\boldsymbol{o}},{\boldsymbol{y}},{\boldsymbol{t}},{\boldsymbol{m}},{\boldsymbol{a}}}}$$

### Sensitivity analysis

We conducted a sensitivity analysis using Monte Carlo simulations to draw random deviates for four model inputs or parameters that affect both the biomass and numbers of Chinook salmon consumed: pinniped abundance (${\boldsymbol{N}}{\boldsymbol{)}}$, Kleiber multipliers (${\boldsymbol{\alpha }}$), diet fraction (*FEC*), and salmon condition factor (*CCOND*; the intercept parameter of 0.000011 in O’Neill *et al*.’s^62^ formula relating fish length to energetic content). We also tested sensitivity to two parameters that affect only the calculation of the number of Chinook salmon consumed: fraction of Chinook salmon in the pinnipeds diets that are juveniles (*SEL*) and the length of the juveniles when they enter the ocean areas (*SMTL*). We did not vary killer whale abundance, since it is known with near perfect detection^42,45,63^, and we did not vary the fraction of juvenile Chinook salmon in killer whale diets (killer whales do not consume juveniles).

Random deviations from the mean input values were assumed to be log-normally distributed for all the variables with a standard deviation of 0.1 on the log-scale for variables related to both biomass and numbers of Chinook salmon consumed (*N*, *FEC*, *CCOND*), a standard deviation of 0.05 for the activity multiplier (${\boldsymbol{\alpha }}$), and a standard deviation of 0.5 on the log-scale for inputs related to strictly numbers consumed (*SEL* and *SMTL*). The random deviates for *FEC* and *SEL* were constrained to be between zero and one. Within a simulation the same deviate was applied to all values for a particular model input. For instance, the pinniped abundance (harbor seals, California sea lions, and Steller sea lions) would all deviate by the same proportion, and similarly, the diet fractions would all deviate by another proportion.

## Electronic supplementary material

Supplementary Information
