# Supplementary material for: Competing tradeoffs between increasing marine mammal predation and fisheries harvest of Chinook salmon
Source: Sci Rep. 2017 Nov 20;7:15439. doi: 10.1038/s41598-017-14984-8 (PMC5696463; doi:10.1038/s41598-017-14984-8)
Supplement: Supplementary file 1 — Supplementary Information [file 41598_2017_14984_MOESM1_ESM.pdf]

Competing tradeoffs between increasing marine mammal predation and fisheries harvest of Chinook salmon

Brandon Chasco[1,9], Isaac C. Kaplan[2], Austen C. Thomas[3], Alejandro Acevedo-Gutiérrez[4], Dawn P. Noren[2], Michael J. Ford[2], M. Bradley Hanson[2], Jonathan Scordino[5], Steve Jeffries[6], Kristin N. Marshall[8], Andrew O. Shelton[2], Craig Matkin[10], Brian Burke[7], Eric J. Ward[2]

[1] Contractor to Conservation Biology Division, NOAA NMFS Northwest Fisheries Science Center, National Marine Fisheries Service, National Oceanic and Atmospheric Administration, 2725 Montlake Blvd. East, Seattle, WA 98112, U.S.

[2] Conservation Biology Division, NOAA NMFS Northwest Fisheries Science Center, National Marine Fisheries Service, National Oceanic and Atmospheric Administration, 2725 Montlake Blvd. East, Seattle, WA 98112, U.S.

[3] Smith-Root, Research Division, 16603 NE 50th Avenue, Vancouver WA 98686, U.S.

[4] Department of Biology, Western Washington University, Bellingham WA 98225, U.S.

[5] Makah Fisheries Management, Neah Bay WA 98357, U.S.

[6] Washington Department of Fish and Wildlife, Olympia WA 98501, U.S.

[7] Fish Ecology Division, NOAA NMFS Northwest Fisheries Science Center, National Marine Fisheries Service, National Oceanic and Atmospheric Administration, 2725 Montlake Blvd. East, Seattle, WA 98117, U.S.

[8] Fishery Resource Analysis and Monitoring Division, NOAA NMFS Northwest Fisheries Science Center, National Marine Fisheries Service, National Oceanic and Atmospheric Administration, 2725 Montlake Blvd. East, Seattle, WA 98117, U.S.

[9] Department of Fisheries and Wildlife, Oregon State University, Corvallis, OR 97331, U.S.

[10] North Gulf Oceanic Society, 3430 Main St. Suite B1, Homer, Alaska, 99603

Figure S1. Estimated coastwide biomass of Chinook salmon relative to the average biomass for the base period of 1979 to 1982 (shaded grey region) for each of the eight areas in our model.

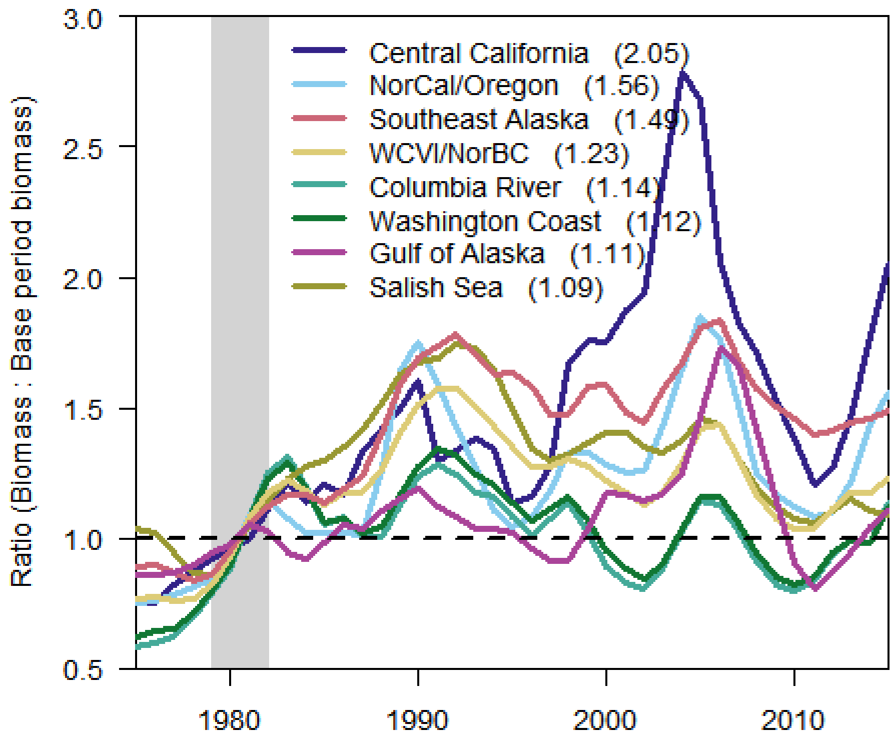

SUPPLEMENTAL MATERIAL DESCRIBING THE DATA FOR THE MODEL

All excel files an r-scripts are available on the github site at https://github.com/bchasco/COAST_WIDE. The model can be run using the buildModel.r wrapper. For each section we describe how the data were compiled, the assumptions that were made, specific files where the data are located, the Excel files related to summaries of the data or data interpolations, and r-scripts that are used to read in the files and build the model. In an attempt to maintain a clean version of the data without any equations, and in an easily readable format, we placed summary tables in a file labeled "appendixTables.xlxs".

Lastly, we constructed a simple bioenergetics model in Excel model ("realityCheck.xlsx") to assist readers and reviewers to understand how the parameters of the model affect the consumption of Chinook salmon. This Excel model doesn't have the temporal or spatial components. It is meant to show how each predator's abundance, mass, residence time in an area, diet fraction and prey size affect the number and biomass of Chinook salmon consumed.

Predator abundance

The variable $N_{p,h,y}$ describes the predator abundance (see Methods section for equations). The summary of the predator abundances can be found in "Table_PredatorAbundance" worksheet in the file appendixTables.xls. The predator abundance time series with model for estimating missing values can be found in predatorAbundanceSources.xlxs file: this file contains data from tables or figures in literature that were used to generate the complete estimates of abundance for all years with and without surveys. The data that are read into the model are found in the predAnnualTotals.csv files. This csv data file is read into the model using the create_Nphy.r R wrapper script.

Killer whales

The annual estimates of killer whale abundances are based on mark-recapture data. The southern resident killer whales (SRKW) in the Salish Sea have perfect detection probability, while the northern resident killer whales (NRKW) along the west coast Vancouver Island/British Columbia coast, southeast Alaska residents (SEAK) in the southeast Alaska area, and the western Alaska residents in the western Alaska area all have imperfect detection in their surveys. For our analysis the individual pods, or family groups, in each population include the following: J, K, and L pods; SRKW [1], +A102, -A11, -A30, -A24, -AC6, -G29, and -I11 pods; NRKW [2], AF, and AG pods; SEAK [3], and AD, AI, AJ, AK, and AN pods; GOA [3,4].

Harbor seals

Harbor seal estimates are derived from time-series of haul-out data. In most cases these time-series have missing years. To interpolate those missing data, we use the MARSS package in R for time-series analysis. The abundance estimates for central California [5,6] are based on the San Francisco Bay and Point Reyes populations. The northern California population of harbor seals is based on the total California population estimate [5] minus the central California estimate.

Columbia River harbor seal estimates are based on annual counts at the mouth of the river. These were digitized from a slide by Robin Brown from ODFW, in lieu of recent comparable estimates.

The Oregon and outer Washington coast both have individual time-series of abundance based on the report by Carretta et al. [5].

The Salish Sea estimate is a combination of federal [5], state [7], and Canadian assessments [8]. These reports include haul-out surveys which must be expanded by a factor of 1.52 to account for seals in the water.

West coast Vancouver Island, coastal British Columbia and Queen Charlotte Island populations are based on Canadian assessments [8]. These surveys are unique from the inland waters because they include both in-water and haul out surveys, so they do not need to be expanded by a correction factor of 1.52 for seals in the water; however, these survey are only a subset of the coast and survey indices needed to be expanded by a factor of 3 for un-surveyed portions of the coastline.

Time series of southeast Alaska and western Alaska populations of harbor seals are very sparse. We used a combination of the most recent Alaska stock assessment reports [9] and time-series of abundance from the literature [10–14] to reconstruct estimates of harbor seal abundance. Complicating this effort is that not all areas were surveyed in every year, and the survey correction factors may not account for the same covariate between studies. Our estimates of Alaska harbor seal abundance allowed rough estimates of the numbers in western and southeastern Alaska. Based on the surveys we collected, we had to: 1) extend the

estimates of abundance beyond the margins of the surveys (i.e., pre-1992 and post-2003), 2) scale the regional estimates of abundance to total estimates of abundance, and 3) partition estimates of abundance into western and southeast Alaska areas.

California sea lion

California sea lion populations are present in model areas 1 to 5 - central California north through the Salish Sea. The abundance estimates for California sea lions are based on pup count surveys conducted by state and federal agencies, and using an expansion factor for pup to adults [15]. The largest concentration of California sea lions is in central California at the major breeding colonies in the Channel Islands and Año Nuevo, California [5]; this is the most complete time-series for California sea lions and these sea lions are considered to be part of the central California population.

Only California sea lions ages 6 and older are likely to prey on Chinook salmon (Jeff Laake, pers. comm. NOAA). However, the time-series of abundance for California sea lions is based on pup counts expansions. Multiplying the time-series by the fraction of the population that is age 6 and older (Jeff Laake, pers. comm NOAA) yielded the number of male and female age 6+ California sea lions. We assume that all age 6+ females remain in Central California, and the number of age 6+ males in Central California is assumed equal to the total number of age 6+ males minus the abundance in the other regions as detailed below.

Based on a single technical report [16], we estimated that the California sea lion population on the outer coast of Washington is 500 individuals, and the Oregon coast population is 4,800 individuals. We have no time series information on these populations, so we assumed that there was no change in abundance for these areas between 1975 and 2015.

Columbia River sea lion estimates are based on a time series of peak counts from a presentation by Robin Brown (pers. comm., Oregon Dept. Fish and Wildlife, 7/29/2015). These peak counts for the Columbia River are only from 2004 to 2015; however, we know that California sea lion were in the

Willamette River starting in the early 1990s [17]. We fit a simple exponential growth model to the peak observation between 2004 and 2015, and then back calculated the number of California sea lions present in the Columbia River between 1990 and 2003 based on the growth rate from the exponential model (see "CSL_ColumbiaRiver" worksheet in the predatorAbundanceSources.xlsx file).

The abundance of California sea lions in the inland waters of the Salish Sea was based on published surveys and estimates [18–20]. We chose to fit a logistic model to the time series of observations based on the single haul-out location at Race Rocks [19]. While the earlier time series of Bigg [18] includes several other haul-out sites in the Strait of Georgia, we chose not to include these observations in our estimates because they do not overlap with later surveys and there is potential for a negative bias in our population estimate. Since the Race Rock survey is for hauled-out sea lions, we use an expansion factor of two to account for sea lions that were in the water during the survey.

We used the time-series of abundance from Bigg [18] to estimate the number of California sea lions along the west coast Vancouver Island/British Columbia area. Again, we fit a logistic model to the available survey data to estimate years with missing data. We did not multiply this estimate by an expansion factor because this survey included both haul-out and in-water (i.e. rafting) observations.

Recently small numbers of California sea lions were observed in Alaskan waters, but we have chosen not to model these based on the short time-series and limited observations [21].

Steller sea lions

While NOAA has produced time series of abundance for the Steller sea lion populations along the eastern Pacific, the spatial resolution of these estimates does not match the spatial areas in the model. We therefore disaggregated many of the NOAA abundance estimates for California, Washington, Oregon, British Columbia, and southeast Alaska. As described below in more detail, we disaggregated the California population into central and northern California, and we estimated a time-series of abundance for the Salish Sea based by disaggregating the British Columbia/west coast Vancouver Island and outer

Washington Coast populations. We also derived estimates of Steller sea lion abundance specifically for the Columbia River and Gulf of Alaska.

We divided Steller sea lion abundance in California into northern and central California regions, using analyses by Pitcher et al. [22] and Angliss and Allen [15]. Based on the figures in Pitcher et al. [22], the ratio between the numbers at Año Nuevo (central California) and the numbers at St. George reef (northern California) has steadily decreased. We digitized the plot in Pitcher et al. [22] and fit exponential curves to the haul out data for each area. We then used the curves to estimate the ratio of central and northern California Steller sea lion populations over time (see the "SSLinterpolated" worksheet in predatorAbundanceSources.xlsx for ratios).

The Gulf of Alaska population estimates were based on survey estimates from Sease et al. [23] and Fritz et al. [24], and unpublished data by Trites and Donnelly[25]. We used a linear interpolation between population estimates for 1960, 1977, 1985, 1989, 1990, 1991, 1992, 1994, 1996, 1998, 2000, and 2002. We placed the analysis of the Gulf of Alaska Steller sea lion numbers in a separate worksheet titled, "SSL_EGULF" in the predatorAbundanceSources.xlsx file.

The population of Salish Sea Steller sea lions are mostly likely transient males that migrate from coastal British Columbia and the Washington Coast to inland waters during the fall, winter and spring months [18,20]. Because their population numbers are low relative to other areas, it was easier from a modeling perspective to treat these individuals as if they were a separate Salish Sea population; within the model, during the summer months when they are not present they are distributed evenly along the Washington Coast and outer British Columbia coast. We found three surveys of Steller sea lion abundance in the Salish Sea: Bigg [18], Edgell and Demarchi [19], and Jefferies et al. [20]. During the 1970s Bigg observed approximately 35-330 Stellers sea lions in British Columbia inland waters, Jefferies observed peaks counts of ~350 in Washington State inland waters, and Edgel and Demarchi observed haul-outs of 680 individuals at Race Rocks in the Strait of Juan de Fuca. Similar to the California sea lion

estimates for the Salish Sea, we fit a logistic model to the Edgell and Demarchi time-series because it was the most complete of the three surveys, with the understanding that using just Race Rocks surveys probably represents an underestimate of Steller Sea lion abundance. We then multiplied the haul-out estimates by a correction factor of two to account for sea lions in the water.

We have no time-series of abundance estimates for the Columbia River. A table in Wiles [26] describing the range of maximum numbers in the Columbia River between 1976 and 2014 is our best source of information. Based on the range given in Wiles, we simply assumed that Steller sea lion have a time-series of abundance that is equal to California sea lions described by Brown.

Predator age and sex structure

This section describes the variables related to predator age structure, $PA_{p,h,y,i}$, and predator sex ratio, $PF_{p,h,y,i}$ – specifically, the percentage of females at each age. A brief summary of the age and sex ratios of the predators is contained in the worksheets "Table_PredatorAgeRatios" and "Table_PredatorFemaleRatios" in the appendixTable.xlsx file. The estimated data for each species that is imported into the model can be found in predatorAgeRatios.csv and predatorFemaleRatio.csv. They are read into the model with the create_Nphyis.r wrapper.

The sex and age distributions of the southern and northern resident killer whales in the Salish Sea [1,27] and west coast Vancouver Island/British Columbia [28] are based on small populations with a high percentage of marked and recapture individuals. The sex and age distributions of these populations are known with very little error. Southeast and western Alaska residents are based on stage-based estimates from Matkin et al. [4]. Without additional information on survivorship, we evenly distributed that fraction of the population in each stage across all ages within a stage.

The sex ratios for seal and sea lions are known with less precision. We used sex and age-specific survivals for harbor seal [29], California sea lion [30], and Steller sea lion [31], and fecundities (harbor seals [32],

California sea lions [30,33], and Steller sea lions [34]) to estimate the stable age distributions of male and female pinnipeds (see the excel file PinnipedStableAgeDistributions.xlsx for projections of the stable age distribution).

Based on input from Jeff Laake at the NOAA Marine Mammal Laboratory (*pers. comm* , 12/19/2016), only California sea lions age 6 and older in any of the areas are likely to be preying on Chinook salmon. Therefore, after determining the stable age distribution for California sea lions, we normalized the ratios to include only those ages six and older.

For some predators like California and Steller sea lion in the Columbia River and the Salish Sea, and California sea lions along the outer coasts of Washington and Oregon, the populations are dominated by sub-adult and adult males. In those instance, the ratio of females in those populations are set to zero.

PREDATOR MASS

For a summary of the predator weight-at-age see the worksheet "Table_PredatorWeightAtAge" in the appendixTable.xlsx file. The variable in the model is $M_{p,h,i,s}$. The data for the model are in predatorWeightAtAge.csv and the data are read into the model with the create_Mphis.r wrapper.

The predator mass is based on published results in the literature. Killer whale mass-at-age was taken directly from the appendix in Noren [35]. California and Steller sea lion mass-at-age is based on the models in Winship et al. [36]. We were unable to find a mass-at-age relationship for Pacific harbor seals. There is a relationship for Atlantic harbor seals [37], but we were uncertain if these seals were representative of the seals in the Pacific. Instead we used data from Figure 8 on page 269 of Pitcher and Calkins [38] and then fit a Gompertz relationship to the median estimates in the figure.

BIOENERGETIC MULTIPLIERS AND DIGESTIVE EFFICIENCY ESTIMATES

The variables in the model for the Kleiber multiplier and digestive efficiency are $\alpha_{p,i,s}$ and Ef_p, respectively. The Kleiber multipliers for killer whales [35], harbor seals [39,40], California sea lions [41], and Steller sea lions [31] are based on published literature values. Units are inconsistent in this literature: watts, joules or calories are reported. For consistency, we describe all of the Kleiber multipliers in units of (kcal/kg). The data input file for the R model is kleiberAlpha.csv. The efficiency inputs are in the wrapper create_EPphis.r. The details of the Kleiber multiplies and digestive efficiencies of each predator are described below.

Killer whales

The killer whale energetic requirements are based on Noren [35]. Noren produced estimates in kcal/kg so there is no transformation of units. The multiplier is straight forward: it is based on the

average field metabolic rate (FMR) based on equations 1 and 2 in Noren. The digestive efficiency of killer whales is estimated to be 0.847 from Williams et al. [42].

Harbor seals

We apply the Howard et al. [40] bioenergetics model for harbor seals, which is based on Boyd [39]. In the Howard paper there are three activities, each with a separate activity multiplier: rest, diving, and surface in a ratio of approximately (20/40/40). Based on this ratio of activities, and transforming from original units of watts to calories, the average activity multiplier for harbor seals is 103 kcal/day. Based on Howard et al. the combination of digestive and heat loss results in an efficiency of 0.825.

California sea lions

The field metabolic rate multiplier for California sea lions was 305 for at-sea, and 122 on-shore, with the time at-sea and on-shore being about 50/50 for an average FMR of 214 [41]. This estimate is also in kcal/kg so there is no converting from joules. However, this estimate does not account for digestive efficiency which we set equal to 0.875 based on the value for Steller sea lions in Winship et al. [31].

Steller sea lions

The estimate of energetic demands for Steller sea lion is based on Winship et al. [31]. The Winship estimate is in kJ/kg and varies by sex and age. The basal metabolic rate is 293. The metabolic multipliers for on-shore and off-shore activities are 1.2 and 4.0, and the percentage of the time on land is about 30%, versus 70% for off-shore. This gives a rough estimate of 230 for α (i.e., (1.2*0.3+4*0.7)*293 / 4.184(J/cal)). Additionally, we account for digestive efficiency, which is 0.875 [31].

Fraction of the energy derived from Chinook salmon

The fraction of Chinook salmon in the predator diets is described by $FEC_{p,j,t}$ in the methods sections. For a summary of the data see “Table_predatorDietFraction” in the appendixTables.xlsx file.

The data for the model are available in the predatorDietFraction.csv file. The data are read into the model with the create_FECpjt.r wrapper. There are many assumptions to diet fraction estimates, which motivated the broad CVs for diets within our sensitivity analysis.

Killer whales diet information is known with more precision than for pinnipeds, since killer whale diet information is based on direct observation or the genetic analysis of scat. For pinnipeds, the fraction of Chinook salmon in the diet is more uncertain because: 1) most diet data is based on frequency of occurrence data (FO) where the fractions for different prey taxa do not sum to one, and 2) often the FO data are an aggregation of all salmon rather than just Chinook salmon.

We used information from Thomas et al. [43] to transform Chinook salmon FO data into split-sample frequency of occurrence (SSFO) data based on paired observations in their analysis. SSFO calculates average prey occurrence as a fraction across all individual scat samples rather just a frequency of occurrence across scat samples; therefore, the prey fractions sum to one. Thomas et al. [43] found different FO to SSFO correction factors for juvenile (ocean age 0) and adult (ocean age 1+): for juveniles, the correction factor is 0.24, meaning that the SSFO is about one quarter of the FO estimate. The correction factor for adult salmon is 0.16.

The FO diet data were based primarily on as synthesis by Adams et al. [44] which compiled over 300 diets studies for marine mammal predators in the eastern Pacific. Unfortunately, many of the salmon estimates are grouped at the genus level, and we needed estimates of Chinook salmon in the diets. To transform the estimates of aggregate salmon to Chinook salmon, we weighted the FO estimate by the average ratio of Chinook salmon in the sport and recreation catch relative to all salmon in the sport and recreational catch, and assumed that predators have the same selectivity for adult salmon as the fishermen. The ratios of Chinook salmon relative to all salmon are: central California 0.95; Northern California and coastal Oregon, 0.90 (see http://odfw.forestry.oregonstate.edu/spawn/pdf%20files/coho/CoastalCohoESUSpawnHarvestSummary.pdf and Coho technical committee report); Columbia River, 0.58 (http://www.critfc.org/fish-and-

watersheds/columbia-river-fish-species/columbia-river-salmon/); outer Washington coast, 0.30 (see http://wdfw.wa.gov/fishing/harvest/ and Chinook technical committee report). Salish Sea Chinook salmon fraction is highly variable because the odd/even year influence of pink salmon but we assume a ratio of 0.05 [45]; BC coast, 0.04 [45]; southeast Alaska, 0.0006 (see http://www.adfg.alaska.gov/index.cfm?adfg=commercialbyareasoutheast.salmon_harvestbyspecies), and western Alaska; 0.0004 (http://www.adfg.alaska.gov/index.cfm?adfg=commercialbyareasoutheast.salmon_harvestbyspecies). Based on these estimates of average Chinook salmon fraction in the catch, we multiplied any aggregate salmon FO estimates by these correction factors. We recognize that in many instance, the odd and even year fraction of Chinook salmon relative to other salmon species will vary dramatically as a function of the pink salmon returns.

Within our Columbia River region, we create average diet compositions that account for California and Steller sea lion individuals that appear to feed directly at the dam and specialize on salmon, versus individuals that feed on a broader set of prey in the lower Columbia River. We assume that ~7% of each sea lion population in the Columbia River model region feeds directly at the dam, and the other 93% feed in the lower river, based on reports for California Sea Lions [1]. The diets of the 7% of the California sea lions that feed directly at the Bonneville have a diet fraction of about 72% Chinook salmon (Adams et al. 2016), and this is based on direct observation, so no FO correction factor is needed. The diet fraction of 93% of California sea lions in the lower river is 18.1% FO aggregated across all salmon species [44]. This translates into about 10.5% FO after accounting for the fact that Chinook salmon are typically 58% of the total returning salmon. The diet fraction of the Steller sea lion feeding directly at the base of Bonneville dam is 39.4% [44]. In the lower river, Steller sea lion consumption of all salmon species is 25% FO ([44]based on a single study), equivalent to an FO of 14.5% for Chinook salmon.

---

1 http://www.westcoast.fisheries.noaa.gov/publications/protected_species/marine_mammals/pinnipeds/sea_lion_removals/2017/mmpa_section_120_program_review_20170208_final.pdf

Predator age selectivity of Chinook salmon

The variable for selectivity in our model is $SEL_{p,j,t,a}$. A summary of the selectivity data can be found in “Table_AverageSELAcrossMonths” in the appendixTables.xlsx file. The selectivity data are found in predatorAgeSelectivity.csv, and the wrapper to read in and process the data is create_SELpjta_basedOnMaturationSchedule.r .

Because a gram of juvenile Chinook salmon is assumed to have the same caloric value as a gram of adult Chinook salmon, the biomass of Chinook consumed only depends on the fraction in the predator diet. The number of Chinook salmon that are consumed, however, depends on the size of the Chinook salmon consumed because, for instance, a four ocean adult is equal to ~1,400 juvenile smolts.

Killer whales do not eat juvenile smolts and the selectivity of different ages of adult Chinook salmon can be estimated from scale pattern analysis from predation events [46]. Although the analysis by Ford and Ellis [46] focused on Salish sea killer whale diets, we apply this same age selectivity for the other three resident killer whale populations.

The size selectivities for the pinnipeds in our model are based on the synthesis by Adams et al. [44]. The studies in this synthesis differentiate the size selectivity of pinnipeds based on hard parts in the scat, but they only define salmonid sizes as either juvenile or adult. We chose to distribute the fraction of adults proportionally across the age distribution of Chinook salmon returning to each area. A summary of the papers used to estimate the temporal and spatial diets fractions of the predators is provided below.

Supplementary Table S1. Sources used to estimate the age selectivity for killer whales and harbor seals.

Area	Killer whale	Harbor seals
Central California		Scordino [47] found mostly consumption of adult salmon, we assume this to be **1:9** juvenile to adult.
Northern California/ Oregon		Scordino [47] found mostly consumption of adult salmon, we assume this to be **1:9** juvenile to adult.
Columbia River		Scordion [47] found seasonal differences in the **juvenile:adult** composition: Spring **1:2,** Summer **1:1 ,** Fall **0:1**. Browne et al. [48]suggest based on FO data of all skeletal remains that the juvenile:adult ratio was Spring **19:6**, Summer **5:4** , Fall **1:2**. Spring is March-May, Summer is June - Aug, Fall is Sept-Nov, Winter is December - Feb. Without additional information on the Winter diet, we assume no Chinook in the diets
Outer Coast Washington		Without detailed size composition of the harbor seal diets along the coast, we chose to use the same estimate as Thomas et al. [43] from the Salish Sea: Apri **1:2**, May **1:1** , June 3:1, July **1:1**, August **1:1**, Septemberl **1:15**. October **1:5**, November **0:1.** Since there is no information for Dec,Jan,Feb,Mar, we used Nov ratios for Dec and Jan, and Feb, and Apr ratios for March
Salish Sea	Ford and Ellis [46] and Hanson et al. [49] provide age specific estimates of killer whale consumption based on scales, **2% age one, 18% age two, 55% age three, and 25% age four**	Thomas et al. [43] found monthly differences in the **juvenile:adult** composition: Apri **1:2,** May **1:1 ,** June **3:1,** July **1:1,** August **1:1,** Septemberl **1:15.** October **1:5**, November **0:1**,. Since there is no information for Dec,Jan,Feb,Mar, we used Nov ratios for Dec and Jan, and Feb, and Apr ratios for March
Outer Vancouver Island / British Columbia	Ford and Ellis [46] and Hanson et al. [49] provide age specific estimates of killer whale consumption based on scales, **2% age one, 18% age two, 55% age three, and 25% age four**	Because there were no harbor seal size selectivity data for outer Vancouver Island or coastal BC, we chose to use the juvenile:adult ratios from Herreman et al. [50]: **1:2** or **1:3**.
Southeast Alaska	Ford and Ellis [46] and Hanson et al. [49] provide age specific estimates of killer whale consumption based on scales, **2% age one, 18% age two, 55% age three, and 25% age four**	Herreman et al. [50] found that the ratio of juvenile:adult salmon (not Chinook) was **1:2** or **1:3**.
Western Alaska	Ford and Ellis [46] and Hanson et al. [49] provide age specific estimates of killer whale consumption based on scales, **2% age one, 18% age two, 55% age three, and 25% age four**	Because there were no harbor seal size selectivity data for western Alaska, we chose to use the juvenile:adult ratios from Herreman et al. [50]: **1:2** or **1:3**.

Supplementary Table S2. Sources used to estimate the age selectivity for California and Steller sea lions.

Area	California sea lion	Steller sea lion
Central California	Scordino [47] found very little information on the size of the salmon in the CSL diets, but suggests that most of the salmon are adults stolen from nets, **100% adult.**	In the absence of size selectivity for Central California, we chose to use Scordino [47] which indicates that Stellers are only eating **100% adults.**
Northern California/ Oregon	Scordino [47] found very little information on the size of the salmon in the CSL diets, but suggests that most of the salmon are adults stolen from nets, **100% adult.**	Scordino [47] indicate that Stellers are only eating **100% adults.**
Columbia River	Scordino [47] and Stansell et al. [51] suggest that the majority of the Chinook consumption is from adults, **100% adult.**	Based on Stansell [51], there is no indication that Stellars were eating anything but **100% adults.**
Outer Coast Washington	Scordino etl al. [52] found time-varying patterns in **juvenile:adult** ratios for salmon consumption: **1:1** Spring, **1:4** Summer, and **1:4** Fall. Without additional information on the Winter diet, we used the Spring fractions for January and February, and the Fall fractions for December	Scordino etl al. [52] found seasonal differences in the size composition of the juvneile:adult ratio: **6:1** Spring, **1:1** Summer, **4:3** Fall, **26:1** Winter.
Salish Sea	There are no size selectivity studies for the Salish Sea. There are similarities between CSL estimates by Scordino etl al. [52] and HS estimates by Thomas et al. [43]. We chose to use the Outer coast estimates from Scordino etl al. [52]: **1:1** Spring, **1:4** Summer, and **1:4** Fall.	There are no size selectivity studies for the Salish Sea. Scordino etl al. [52] found seasonal differences in the size composition of the juvneile:adult ratio: **6:1** Spring, **1:1** Summer, **4:3** Fall, **26:1** Winter.
Outer Vancouver Island / British Columbia	We found no size selectivity estimates for the Outer Vancouver Island or British Columbia. Given the similarities between harbor seal and California sea lion diets in the Salish Sea and along the outer coast of Washington, respectively. We chose to use the size selectivity of the harbor seals in from Southeast Alaska based on the Herreman et al.[50] study: **1:2** or **1:3**, juvenile to adult.	There are no size selectivity studies for the outer Vancouver Island and coastal BC. Tollit et al. [53] and Sigler et al. [54] estimated the fork length of the salmon in the diets to be **32.5** cm and **31+-12**cm . That is approximately the size of age 1 fish. Without any other information we assumed that all of these were ocean **age 1**.
Southeast Alaska		There are no size selectivity studies for the outer Vancouver Island and coastal BC. Tollit et al. [53] and Sigler et al. [54] estimated the fork length of the salmon in the diets to be **32.5**cm and **31+-12**cm . That is approximately the size of age 1 fish. Without any other information we assumed that all of these were **age 1**.
Western Alaska		Sinclair and Zeppelin [55] found mostly adult size fishes, although salmonids comprised less than 5% FO. We assumed that this meant there were no smolts in the diets and only **100% adult**.

Predator temporal and spatial distributions

The variable in the model that describes the predator distribution is $\Phi_{p,h,j,t,s}$. Data for the model is determined by results found in the literature, or by expert opinion when data are lacking. Each of the predators in our model is assumed to originate from a home area. Throughout the course of the year the different predator populations may disperse based on their sex – male California and Steller sea lions are more mobile. The data that are input into the model are found in the file predatorTemporalSpatialDistribution.csv, and the wrapper that reads in and processes the data is create_PHIphjts.r. For a summary of the data see the worksheet "Table_temporalSpatialPredator" in the appendixTables.xlsx spreadsheet.

Killer whales

Southern resident killer whales occupy Salish Sea waters from May to early November. During that period they are not in Salish Sea waters 100% of the time – even during their peak occupancy in summer months they only occupy inland waters 80% of the time [56,57]. During the summer months we assume that 20% of SRKW are not present in Salish Sea waters, and instead are evenly distributed among the areas from central California to the west coast of Vancouver Island. There are three pods of killer whales in the Salish Sea: J, K, and L. During the winter months J-pod, which represents about 30% of the population, spends about 25% of its time in Salish Sea waters. The remainder of its time is spent evenly between Central California, Northern California and Oregon, and the outer coast of Washington. The K and L pods are assumed to spend their time evenly between Central California, Northern California and Oregon, and the outer coast of Washington, and no time in Salish Sea waters. This means that about 7.5% of the population occupies the waters of the Salish during the fall and winter months.

Northern residents killer whales occupy the waters of west coast Vancouver Island and the British Columbia outer coast 100% of the time [58,59]. During the summer months the northern residents are known to feed in Johnstone Strait at the northern end of Vancouver Island but we do not consider this area to be part of the Salish Sea.

The killer whales in the Gulf Of Alaska (Prince William Sound, Cook Inlet, Kenai, and Kodiak Island) appear more mobile than the other resident populations [60]. Whales from Prince William Sound can be observed in southeast Alaska and off of Kodiak Island [60], and whales from southeast Alaska are known to occupy waters of western Alaska. To reflect the mobility of the southeast resident killer whales we assume they spent 50% of their time in western waters and 50% in southeastern waters. For western residents we simply assume they spend 100% of their time in Gulf of Alaska waters.

Harbor seal

Harbor seals are assumed to be year-round residents in their area of origin, with no exchange between adjacent areas [61]. The one exception is for harbor seals in the Columbia River. We assume these seals migrate to the outer coast during the breeding season – half go to the Washington coast and the other half go to the Oregon coast. Some evidence suggests that harbor seals may migrate more than previously expected. In a recent study Peterson et al. [62] demonstrated two of the 20 seals fitted with satellite tags in Salish Sea waters migrated briefly to the outer coast of Washington during the spring months. However, for the purposes of this study we assume they remain year round residents of their home area.

California sea lion

We assume the population of female California sea lions in central California are year-round populations. From the model perspective it is easier to assume the populations of male California sea lions are resident in the Salish Sea [18,20], Columbia River (Robin Brown pers comm.), and coastal populations of Oregon and Washington, and they migrate to the central California area during their breeding periods in May and June. In addition to the seasonal movements to the breeding colonies in

California, there is a decline in the abundance of sea lions during the winter in the Salish Sea and Columbia Rivers. During this time we assume that the sea lion move to the bays and estuaries along the outer coast of Washington and Oregon.

Steller sea lions

Similar to the California sea lion populations, it is easier to assume the populations of Steller sea lions in the Salish Sea [18,20] and Columbia River [51] (Robin Brown pers comm.) are residents that migrate to adjacent areas during periods of absence. However, since we do not know exactly where they migrate to when they are absent [18] we have chosen to assume that they move to the bays and estuaries on the outer coasts of Washington, Oregon, and Vancouver Island.

CHINOOK SALMON PRODUCTION

The variable that describes the recruitment of Chinook salmon to the areas where predation occurs is $R_{h,r,o,y}$. There are two files containing the raw data related to smolt production:

Smolt_AllData.csv which is the hatchery production, and WildProduction.csv which are the estimates wild production based on the escapement data. The data are read in with the wrapper create_Rhroy.r. A summary of the wild and hatchery production is in the worksheet “Table_SmoltProduction” in the appendixTables.csv file.

There are two sources of Chinook salmon production – hatchery and natural origin fish. Estimates of hatchery production were determined using the RMIS database [63]. There are a number of ways to generate the abundance of outmigrating smolts. We used the “Adclipped Releases” database and selected only Chinook salmon releases. Although the database are labeled Adclipped releases, it includes an estimate of both adclipped and unmarked releases. Using the “release_location_rmis_region” field in the RMIS database we were able to map the release data to the areas in our model (Supplementary Supplementary Table S3).

There is no database for wild smolt production. To estimate the annual smolt production we compiled escapement data for natural spawners in the tributaries along the west coast based on reports [64–66] (Supplementary Supplementary Table S4). With the exception of the Canadian stocks, most of the escapement estimates are available as downloadable Excel files from management websites. In the case of the Canadian stocks we had to digitize the escapement figures based on presentations and papers containing of post season reconstructions [67,68].

There are very few experiments to estimate the number of natural origin juvenile produced. To estimate natural origin smolts we multiply the estimated escapement by 0.5 to estimate the number of female spawners, and then multiply the number of female spawners by the average number of juveniles-per-female. The estimated average number of juveniles-per-females was 220 based on a combination of sources in Supplementary Supplementary Table S5, and a review of Columbia River production in Petrosky et al. [69].

Supplementary Table S3. Lookup table for the relationship between the area names in our model and the regional tag code identifiers for hatchery released Chinook salmon in the RMIS database.

Area ID	Area name	release_location_rmis_region
1	Central California	CAGN, CECA, SAFA, SJOA
2	Northern California/Oregon	KLTR, NOCA, NOOR, ORGN, SOOR
3	Columbia River	CECR, CRGN, LOCR, SNAK, UPCR
4	Outer Washington Coast	GRAY, NWC, WAGN, WILP
5	Salish Sea	FRTH, GST, HOOD, JNST, JUAN, MPS, NOWA, NPS, QCI, SKAG, SPS
6	West Coast Vancouver Island/Coastal British Columbia	COBC, NASK, WCVI
7	Southeast Alaska	ALSR, CHIL, SEAK, STUN, TAWH, TRAN
8	Western Alaska	WEAK

Supplementary Table S4. Tributaries in each area and estimate average escapement based on Chinook Technical Committee reports, Pacific Salmon Commission Appendix A report, and presentations by Department of Fisheries and Oceans in Canada. In some instances reports group escapement estimates (e.g., above Bonneville, west coast Vancouver Island index streams, etc.).

Area ID	Area name	Watersheds	Average escapement
1	Central California	San Joaquin, Sacramento	151,431
2	Northern California/Oregon	Klamath/Trinity, Oregon North Coast, Oregon South Coast, Shasta Area, Rogue, Rogue/Umpqua	173,642
3	Columbia River	Above Bonneville, below Bonneville, Mid-Columbia Brights, Up-River Brights	323,297
4	Outer Washington Coast	Grays Harbor, Hoh, Queets, Quilyutte, Quinalt, Willapa Bay	31,045
5	Salish Sea	Fraser, Hood Canal, Puget Sound, Skagit, Stillaquamish/Snohonmish	91,022
6	West Coast Vancouver Island/Coastal British Columbia	West coast Vancouver Island aggregate, Nass, Skeena	76,215
7	Southeast Alaska	Copper, Stikine, Skeena	93,982
8	Western Alaska	Chignik, Karluk, Kenai, Susitna	75,612
Total			1,016,248

Supplementary Table S5. Natural juvenile production estimates based on a review of the available literature.

Source	Tributary	Run type	Estimate and page number
Kiefer et al. [70]	Snake River	Spring/Summer	243
Zimmerman et al. [71]	Skagit River	Spring, summer/fall	750, page 8
McPherson [72]	Taku River	Spring/Summer	90, page 28
Seiler et al. [73]	Green River		90, page 2-16
Seiler et al. [73]	Chiwawa	Sping	233, page 3-17 yearling only
Carmichael et al. [74]	Upper Grande Ronde	Spring	80, page 15
Kinsel et al. [75]	Skagit River	Spring, summer/fall	218, page 26
Kiyohara and Volkhardt [76]	Bear creek	Fall ?	303, page 43
Kiyohara and Volkhardt [76]	Cedar	Fall ?	703, page 28
Lamperth et al. [77,78]	Coweeman	Fall	560
Seiler et al. [79]	Cedar	Fall ?	479, page 38
Seiler et al. [79]	Bear creek	Fall ?	95, page 53

CHINOOK CATCH

Estimates of Chinook salmon commercial and recreational catch are summarized in the Excel file HarvestFromPMFC.csv. The file includes data from three sources: Pacific Fishery Management Council [66], Pacific Salmon Commission Joint Technical Committee [65], and the Alaska Department of Fish and Game website for commercial salmon catches [80], depending on the area and year when the data were available. The specific tables where the data were taken from are described in the worksheet. To get specific catches for the Gulf of Alaska you will need to query the individual years from the dropdown menu on the website.

CHINOOK ENERGY CONTENT

The variable related to Chinook salmon energy in our model is $EC_{h,r,o,t,a}$. The energy of Chinook salmon was estimated as a function of its length, and the data on Chinook length were derived from release data in the RMIS database for coded-wire-tagged (CWT) fish. Our assumption was that wild and hatchery fish are about the same size, and the average size of release fish by area and run type reflects the mixture of sub-yearling and yearling fish in the populations. The raw length data for the model are in Smolt_AllData.csv for the juveniles, and ChinookLengthAtAge.csv for the adults. The wrappers to create the length-at-age arrays are create_SMTLhryt.r and create_CLAmrota.r. A summary of the length at data is available in the worksheet “Table_ChinookLengthAtAge” in the appendixTable.xlsx file. Lastly, the wrapper to transform the length-at-age of Chinook salmon into energy-at-age of Chinook salmon is in create_EChromta.r.

We used the energy model developed by O’Neill et al. [81] to estimate the kcal of Chinook salmon energy as a function of Chinook salmon length. Smolt length is based on average length of smolt releases from the RMIS database. We used a separate database than the one that was used to estimate the hatchery total production. We used the same regional codes in Supplementary Supplementary Table S3 to map the

hatchery release locations to the areas in our model. Our estimates of smolt length did not vary across years, but they did vary by area, run type, and across months.

In many cases there may have been no recorded smolt lengths for a particular area and run. When that occurred we took the average of the smolts lengths from the adjacent areas by run type, and if there were no observations for a run in the adjacent areas we took that average across all observed runs in the adjacent areas.

Based on work by Teel et al. [82] and Weitkamp et al. [83] we expect the juvenile Chinook salmon to stay in the coastal areas for more than one model time-step. Rather than model the residence of juvenile Chinook salmon in each area, we assumed that the size of the juveniles was equal to the release size of the juvenile, plus 40 days of growth – 10 days for the estimated migration time between the release site in the rivers, or rearing location for natural stocks, and an additional 30 days for the average residence time in each area. Based on work by Weitkamp et al. [83] we used an average of 1.0 mm/day in growth for all run types.

Monthly estimates of adult length-at-age were based on model estimates from the FRAM model [84]. These estimates are for specific river systems in each area. To get an aggregate estimate adult size within an area, we simply averaged across all rivers within an area – ignoring any weighting based on the relative abundance of fish returning for the different rivers. The FRAM model did not have estimates of the length of ocean age 5 Chinook salmon, so we projected the von Bertalanffy model forward based on average growth parameters across all rivers (Linf equals 1146, K equals 0.0305, and t0 equals -2.79) and estimated the average length of an ocean age five individual. Note the parameters of the FRAM model are for a monthly time step, so the age of five ocean fish is 72 months, not 5 years.

Chinook salmon distribution

The variable $\Theta_{h,j,r}$ describes the spatial distribution of the Chinook salmon in our model. A summary of the distribution can be found in the worksheet "Table_ChinookTempSpatialDist" in the appendixTables.xlsx file. The raw data are in the WeitkampDistribution.csv and they are read into the model using the wrapper create_THETAhjromta.r.

The distribution of adult Chinook salmon is based on the study by Weitkamp [85]. To map the areas in Weitkamp [85] to the areas in our model we used look-up Supplementary Supplementary Table S6. The distribution of smolts assumes that they are only available to predation during the month they entered freshwater in their area of origin, after which they entered a black box where they were unavailable to predators until the beginning of the next year.

Each area and run has a unique distribution but not all of the runs and areas in our model were represented by a particular stock in the Weitkamp [85] results. To interpolate missing information we used the following rule based on discussions with Weitkamp (pers comm). : 1) spring and summer Chinook salmon tended to disperse further north than fall Chinook salmon, 2) fall Chinook salmon tended to remain near their area of origin and their distributions closely resembled neighboring areas, 3) recoveries of hatchery and wild fish had similar marine distributions, and 4) ocean age 1 fish were captured closer to the area of origin compared to older aged fish. Based on these findings, if either spring or summer distributions for a particular area were missing, we assumed that spring and summer run distributions were the same and used which ever one was available.. If both the spring and summer runs were missing, we averaged the spring and summer runs from the closest adjacent area that had available tag information. For areas with missing fall Chinook salmon tag information (e.g., mainland British Columbia to western Alaska), we assumed that 80% of the stock was distributed in the area of origin, and 10% was distributed to adjacent areas. We assumed that winter Chinook salmon populations had the same distribution as fall Chinook salmon, and we assumed the "other" category, which is an aggregate of

several stocks into a single release group, was an average across all observed and interpolated distributions.

Supplementary Table S6. Lookup table for area IDs in our model and the hatchery IDs in Weitkamp (2010).

Area ID	Hatchery ID	
Cen California	COLEMAN NFH Fall	TEHAMA-COLUSA FF Fall
	FEATHER R HATCHERY Fall	
Nor. Cal/Oregon	COLE RIVERS HATCHERY Fall	SALMON R HATCHERY Fall
	COLE RIVERS HATCHERY Spri	TRASK R HATCHERY Fall
	ELK R HATCHERY Fall	TRINITY R HATCHERY Fall
	IRON GATE HATCHERY Fall	TRINITY R HATCHERY Spri
	ROCK CR HATCHERY Spri	
Columbia River	ABERNATHY SCDC HATCH Fall	PRIEST RAPIDS HATCHE Fall
	BONNEVILLE HATCHERY Fall	SIMILKAMEEN HATCHERY Summ
	COWLITZ SALMON HATCH Fall	SPRING CR NFH Fall
	COWLITZ SALMON HATCH Spri	STAYTON PD (WILLAMET Fall
	KLICKITAT HATCHERY Fall	WASHOUGAL HATCHERY Fall
	LYONS FERRY HATCHERY Fall	Wells H/Sp Chan Summ
	MARION FORKS HATCH Spri	WILLAMETTE HATCHERY Spri
	MCKENZIE HATCHERY Spri	BONNEVILLE HATCHERY Late
outer Washington	FORKS CREEK HATCHERY Fall	IRRIGON HATCHERY Late
	HOKO FALLS HATCHERY Fall	Quinault Lk & NFH Fall
	HUMPTULIPS HATCHERY Fall	SOLDUC HATCHERY Spri
Salish Sea	ELWHA HATCHERY Fall	HOODSPORT HATCHERY Fall
	Fraser abv Hope Spri	H-PUNTLEDGE R Fall
	GROVERS CR HATCHERY Fall	H-PUNTLEDGE R Summ
	H-BIG QUALICUM R Fall	H-QUESNEL R Summ
	H-CAPILANO R Fall	H-QUINSAM R Fall
	H-CHEHALIS R Fall	H-ROBERTSON CR Fall
	H-CHEMAINUS R Fall	H-SHUSWAP R Summ
	H-CHILLIWACK R Fall	H-SPIUS CR Spri
	H-CLAYOQUOT Fall	H-TENDERFOOT CR Summ
	H-CLEARWATER R UP/TO Summ	ISSAQUAH HATCHERY Fall
	H-CONUMA R Fall	KENDALL CR HATCHERY Spri
	H-COWICHAN R Fall	MARBLEMOUNT HATCHERY Spri
	H-EAGLE R Summ	SAMISH HATCHERY Fall
	H-GOLD R Fall	SOOS CREEK HATCHERY Fall
	H-NANAIMO R Fall	WALLACE R HATCHERY Summ
	H-NITINAT R Fall	
WCVI/ BC	H-FORT BABINE Summ	H-OWEEKENO Summ
	H-KINCOLITH R Spri	H-SNOOTLI CR Summ
	H-KITIMAT R Spri	H-TERRACE Summ
	H-MASSET Summ	
Southeast AK	CRYSTAL LAKE Spri	NEETS BAY Spri
	DEER MOUNTAIN Spri	SNETTISHAM Spri
Western AK	Cook Inlet Spri	

Chinook salmon maturity-at-age and survival-at-age

The variables in the model for maturity-at-age and survival are $MAT_{h,r,o,m,a}$ and $surv_{t,a}$, respectively. The raw data are located in the files FRAM_maturityAtAge.csv and FRAM_survivalRates.csv, respectively, and the data from these files are read into the model with the wrappers create_MAThra.r, create_NCphjromyta.r, respectively. Summaries of the maturity and survival are in the worksheets “Table_maturityAtAge” and “Table_survivalAtAge” in the appendixTables.xlsx files. Note that the numbers in Table_survivalAtAge are actually mortality estimates that are then transformed into survival estimates in the code.

In addition to predation, salmon are removed from the system through natural mortality and escapement. Estimates of maturity- and survival-at-age are based on FRAM model inputs. The mortality estimates from the FRAM model are only month and age-specific: all run types, origins, and salmon from different areas have the same survival rates. The maturation schedules are also based on the 78 distinct stocks FRAM model. The FRAM model is dominated by stocks from Puget Sound, and lacks any stocks from western or Southeast Alaska. For the western and southeast Alaska areas, we used the average maturation schedules from coastal British Columbia.

Relationship between Chinook salmon energy, length, and mass

One of the key aspects of our model is transforming between energy as a function of length, and weight as a function of length or energy. The two key outcomes of our model are the number and biomass of Chinook salmon consumed. We know the length of Chinook salmon at different ages based on the FRAM model [84]. From O’Neill [81] we can use the equation in Figure 6, $E = 0.000011 \times length^{3.12}$, to determine the energy of a Chinook salmon at a given length. Dividing the fraction of predator’s total energy derived from Chinook salmon of particular age by the energy

content of Chinook salmon from that age yields the number of salmon consumed. To obtain an estimate of biomass consumed we use Equation 7 from Figure 3, $Mass = \exp[(\ln(E) - 7.56)/0.94]$. There is a second option for transforming energy into mass using the Equations in Figure 7; however, since we used a stock-aggregated relationship between length and energy, we felt it was appropriate to also use a stock-aggregate relationship between mass and energy.

References

1. Center for Whale Research. Study of Southern Resident Killer Whales. *Center for Whale Research - Study of Southern Resident Killer Whales* (2016). Available at: http://www.whaleresearch.com/. (Accessed: 13th March 2016)
2. Olesiuk, P. F., Ellis, G. M. & Ford, J. K. *Life history and population dynamics of northern resident killer whales (Orcinus orca) in British Columbia*. (Canadian Science Advisory Secretariat, 2005).
3. Allen, B. M. & Angliss, R. P. *Killer whale (Orcinus orca): Eatern North Pacific Alaska Resident Stock*. 9 (NOAA, 2013).
4. Matkin, C. O., Ward Testa, J., Ellis, G. M. & Saulitis, E. L. Life history and population dynamics of southern Alaska resident killer whales (Orcinus orca). *Mar. Mammal Sci.* **30,** 460–479 (2014).
5. Carretta, J. V. *et al.* US PACIFIC DRAFT MARINE MAMMAL STOCK ASSESSMENTS: 2015. (2015).
6. Codde, S. & Allen, S. Pacific harbor seal (Phoca vitulina richardsi) monitoring at Point Reyes National Seashore and Golden Gate National Recreation Area. *2012 Annu. Rep. Available Httpwww Sfnps Orgdownloadprod Uct44500 Accessed May* **2,** (2015).
7. Jeffries, S., Huber, H., Calambokidis, J. & Laake, J. Trends and status of harbor seals in Washington State: 1978-1999. *J. Wildl. Manag.* 207–218 (2003).
8. Canadian Science Advisory Secretariat. *Population assessment: Pacific harbour seal (Phoba vitulina Richardsi)*. (Fisheries and Oceans Canada, 2010).
9. Muto, M. M. *et al. Alaska Marine Mammal Stock Assessments, 2015*. 309 (NOAA, 2016).
10. Boveng, P. L. *et al.* The abundance of harbor seals in the Gulf of Alaska. *Mar. Mammal Sci.* **19,** 111–127 (2003).
11. Small, R. J., Pendleton, G. W. & Pitcher, K. W. Trends in abundance of Alaska harbor seals, 1983–2001. *Mar. Mammal Sci.* **19,** 344–362 (2003).

12. Jemison, L. A., Pendleton, G. W., Wilson, C. A. & Small, R. J. Long-term trends in harbor seal numbers at Tugidak Island and Nanvak Bay, Alaska. *Mar. Mammal Sci.* **22,** 339–360 (2006).

13. Mathews, E. A. & Pendleton, G. W. Declines in harbor seal (Phoca vitulina) numbers in Glacier Bay national park, Alaska, 1992–2002. *Mar. Mammal Sci.* **22,** 167–189 (2006).

14. Womble, J. N. *et al.* Harbor seal (Phoca vitulina richardii) decline continues in the rapidly changing landscape of Glacier Bay National Park, Alaska 1992–2008. *Mar. Mammal Sci.* **26,** 686–697 (2010).

15. Angliss, R. P. & Allen, B. M. *Alaska marine mammal stock assessments, 2014*. 313 (NOAA, 2015).

16. National Marine Fisheries Service (NMFS). *Investigation of Scientific Information on the Impacts of California Sea Lions and Pacific Harbor Seals on Salmonids and on the Coastal Ecosystems of Washington, Oregon, and California*. 172 (1997).

17. Wright, B. E., Brown, R. F. & Murtagh, T. *WILLAMETTE FALLS PINNIPED MONITORING PROJECT, 2015*. (Oregon Department of Fish and Wildlife, 2015).

18. Bigg, M. A. *Status of the Steller sea lion (Eumetopias jubatus) and California sea lion (Zalophus californianus) in British Columbia*. (Department of Fisheries and Oceans Ottawa, 1985).

19. Edgell, T. C. & Demarchi, M. W. California and Steller sea lion use of a major winter haulout in the Salish Sea over 45 years. *Mar. Ecol. Prog. Ser.* **467,** 253–262 (2012).

20. Jeffries, S. *et al. Aerial Surveys of Pinniped Haulout Sites in the PNW; Marine Mammal Aerial Surveys Conducted in the PNW, IPSW; and Offshore Large Whale Satellite Tagging in the NWTRC*. 174 (Naval Facilities Engineering Command, Northwest (NAVFAC NW), 2014).

21. Maniscalco, J. M. *et al.* The occurrence of California sea lions (Zalophus californianus) in Alaska. *Aquat. Mamm.* **30,** 427–433 (2004).

22. Pitcher, K. W. *et al.* Abundance and distribution of the eastern North Pacific Steller sea lion (Eumetopias jubatus) population. *Fish. Bull.* **105,** 102–116 (2007).

23. Sease, J. L., Taylor, W. P., Loughlin, T. R. & Pitcher, K. W. Aerial and land-based surveys of Steller sea lions (Eumetopias jubatus) in Alaska, June and July 1999 and 2000. *NOAA Tech. Memo. NMFS-AFSC* **122,** 52pp (2001).

24. Fritz, L., Lynn, M., Kunisch, E. & Sweeney, K. Aerial, ship and land-based surveys of Steller sea lions (Eumetopias jubatus) in Alaska, June and July 2005–2007. *NOAA Tech. Memo. NMFS-AFSC* **183,** (2008).
25. Trites, A. W. & Donnelly, C. P. The decline of Steller sea lions Eumetopias jubatus in Alaska: a review of the nutritional stress hypothesis. *Mammal Rev.* **33,** 3–28 (2003).
26. Wiles, G. J. *Periodic Status Review for the Steller Sea Lion*. 57 (Washington Department of Fish and Wildlife, 2015).
27. Ward, E. J., Holmes, E. E. & Balcomb, K. C. Quantifying the effects of prey abundance on killer whale reproduction. *J. Appl. Ecol.* **46,** 632–640 (2009).
28. Ward, E. J. *et al.* Long-distance migration of prey synchronizes demographic rates of top predators across broad spatial scales. *Ecosphere* **7,** (2016).
29. Bigg, M. A. *The harbour seal in British Columbia*. (Fisheries Research Board of Canada Ottawa, 1969).
30. Hernández-Camacho, C. J., Aurioles-Gamboa, D. & Gerber, L. R. Age-specific birth rates of California sea lions (Zalophus californianus) in the Gulf of California, Mexico. *Mar. Mammal Sci.* **24,** 664–676 (2008).
31. Winship, A. J., Trites, A. W. & Rosen, D. A. A bioenergetic model for estimating the food requirements of Steller sea lions Eumetopias jubatus in Alaska, USA. *Mar. Ecol. Prog. Ser.* **229,** 291–312 (2002).
32. Härkönen, T. & Heide-Jørgensen, M.-P. Comparative life histories of East Atlantic and other harbour seal populations. *Ophelia* **32,** 211–235 (1990).
33. French, S. S., González-Suárez, M., Young, J. K., Durham, S. & Gerber, L. R. Human disturbance influences reproductive success and growth rate in California sea lions (Zalophus californianus). *PLoS One* **6,** e17686 (2011).
34. York, A. E. The population dynamics of northern sea lions, 1975-1985. *Mar. Mammal Sci.* **10,** 38–51 (1994).

35. Noren, D. P. Estimated field metabolic rates and prey requirements of resident killer whales. *Mar. Mammal Sci.* **27,** 60–77 (2011).

36. Winship, A. J., Trites, A. W. & Calkins, D. G. Growth in body size of the Steller sea lion (Eumetopias jubatus). *J. Mammal.* **82,** 500–519 (2001).

37. Boulva, J., McLaren, I. A. & others. *Biology of the harbor seal, Phoca vitulina, in eastern Canada*. (Dept. of Fisheries and Oceans, 1979).

38. Pitcher, K. W. & Calkins, D. G. *Biology of the harbor seal, Phoca vitulina richardsi, in the Gulf of Alaska*. (Outer Continental Shelf Environmental Assessment Program, US Department of Interior, Bureau of Land Management, 1979).

39. Boyd, I. L. Estimating food consumption of marine predators: Antarctic fur seals and macaroni penguins. *J. Appl. Ecol.* **39,** 103–119 (2002).

40. Howard, S., Lance, M. M., Jeffries, S. J. & Acevedo-Gutiérrez, A. Fish consumption by harbor seals (Phoca vitulina) in the San Juan Islands, Washington. *Fish. Bull.* **111,** 27 (2013).

41. Weise, M. J. & Harvey, J. T. Temporal variability in ocean climate and California sea lion diet and biomass consumption: implications for fisheries management. *Mar. Ecol. Prog. Ser.* **373,** 157–172 (2008).

42. Williams, T. M., Estes, J. A., Doak, D. F. & Springer, A. M. Killer appetites: assessing the role of predators in ecological communities. *Ecology* **85,** 3373–3384 (2004).

43. Thomas, A. C., Nelson, B., Lance, M. M., Deagle, B. & Trites, A. Harbour seals target juvenile salmon of conservation concern. *Can. J. Fish. Aquat. Sci.* (2016).

44. Adams, J. *et al.* A century of Chinook salmon consumption by marine mammal predators in the Northeast Pacific Ocean. *Ecol. Inform.* **34,** 44–51 (2016).

45. Henderson, M. A. & Graham, C. C. History and status of Pacific salmon in British Columbia. *North Pac. Anadromous Fish Comm. Bull.* **1,** 13–22 (1998).

46. Ford, J. K. & Ellis, G. M. Selective foraging by fish-eating killer whales Orcinus orca in British Columbia. *Mar. Ecol. Prog. Ser.* **316,** 185–199 (2006).

47. Scordino, J. *West coast pinniped program investigations on California sea lion and Pacific Harbor seal impacts on salmonids and other fishery resources*. 106 (Pacific States Marine Fisheries Commission, 2010).

48. Browne, P., Laake, J. L. & DeLong, R. L. Improving pinniped diet analyses through identification of multiple skeletal structures in fecal samples. *Fish. Bull.* **100,** 423–433 (2002).

49. Hanson, Mb. *et al.* Species and stock identification of prey consumed by endangered southern resident killer whales in their summer range. *Endanger. Species Res.* **11,** 69–82 (2010).

50. Herreman, J. K., Blundell, G. M. & Ben-David, M. Evidence of bottom-up control of diet driven by top-down processes in a declining harbor seal Phoca vitulina richardsi population. *Mar. Ecol. Prog. Ser.* **374,** 287–300 (2009).

51. Stansell, R. J., Gibbons, K. M. & Nagy, W. T. *Evaluation of pinniped predation on adult salmonids and other fish in the Bonneville Dam tailrace, 2008-2010*. (US Army Corps of Engineers, Bonneville Lock and Dam, 2010).

52. Scordino, J., Akmajian, A. M. & Riemer, S. D. *California and Steller sea lion diets in northwest Washington, 2010-2013*. 218 (2014).

53. Tollit, D. J., Wong, M. A. & Trites, A. W. Diet composition of Steller sea lions (Eumetopias jubatus) in Frederick Sound, southeast Alaska: a comparison of quantification methods using scats to describe temporal and spatial variabilities. *Can. J. Zool.* **93,** 361–376 (2015).

54. Sigler, M. F. *et al.* Steller sea lion foraging response to seasonal changes in prey availability. *Mar. Ecol. Prog. Ser.* **388,** 243–261 (2009).

55. Sinclair, E. H. & Zeppelin, T. K. Seasonal and spatial differences in diet in the western stock of Steller sea lions (Eumetopias jubatus). *J. Mammal.* **83,** 973–990 (2002).

56. Hauser, D. D., Logsdon, M. G., Holmes, E. E., VanBlaricom, G. R. & Osborne, R. W. Summer distribution patterns of southern resident killer whales Orcinus orca: core areas and spatial segregation of social groups. *Mar. Ecol.-Prog. Ser.*- **351,** 301 (2007).

57. National Marine Fisheries Service. *Recovery Plan for Southern Resident Killer Whales (Orcinus orca)*. (2008).

58. Barrett-Lennard, L. G., Heise, K., Saulitis, E., Ellis, G. & Matkin, C. The impact of killer whale predation on Steller sea lion populations in British Columbia and Alaska. *Rep. North Pac. Univ. Mar. Mammal Res. Consort. Univ. Br. Columbia Vanc. BC Can.* (1995).

59. Nichol, L. M. & Shackleton, D. M. Seasonal movements and foraging behaviour of northern resident killer whales (Orcinus orca) in relation to the inshore distribution of salmon (Oncorhynchus spp.) in British Columbia. *Can. J. Zool.* **74,** 983–991 (1996).

60. Matkin, C. O., Ellis, G., Olesiuk, P. & Saulitis, E. Association patterns and inferred genealogies of resident killer whales, Orcinus orca, in Prince William Sound, Alaska. *Fish. Bull.* **97,** 900–919 (1999).

61. Suryan, R. M. & Harvey, J. T. TRACKING HARBOR SEALS (PHOCA VITULINA RICHARDSI) TO DETERMINE DIVE BEHAVIOR, FORAGING ACTIVITY, AND HAUL-OUT SITE USE. *Mar. Mammal Sci.* **14,** 361–372 (1998).

62. Peterson, S. H., Lance, M. M., Jeffries, S. J. & Acevedo-Gutiérrez, A. Long distance movements and disjunct spatial use of harbor seals (Phoca vitulina) in the inland waters of the Pacific Northwest. *PloS One* **7,** e39046 (2012).

63. RMIS. *Regional Mark Information System User Guide*. 79 (Pacific Fishery Management Council, 2012).

64. Department of Fisheries and Oceans. 2014 Fraser River Stock Assessment and Fishery Summary Chinook, Coho, and Chum. (2014).

65. PACIFIC SALMON COMMISSION JOINT SALMON. *ANNUAL REPORT OF CATCH AND ESCAPEMENT FOR 2015*. 225 (Pacific Salmon Comission, 2016).

66. PFMC. *Review of 2015 Ocean Salmon Fisheries: Stock Assessment and Fishery Evaluation Document for the Pacific Coast Salmon Fishery Management Plan*. (Pacific Fishery Management Council, 2016).

67. DFO. *Fraser River Chinook Salmon*. (1999).

68. Heard^1, W. R., Shevlyakov, E., Zikunova, O. V. & McNicol, R. E. Chinook salmon–trends in abundance and biological characteristics. *Bull. No* **4,** 77–91 (2007).

69. Petrosky, C., Schaller, H. & Budy, P. Productivity and survival rate trends in the freshwater spawning and rearing stage of Snake River chinook salmon (Oncorhynchus tshawytscha). *Can. J. Fish. Aquat. Sci.* **58,** 1196–1207 (2001).

70. Kiefer, R. B., Bunn, P. R. & Johnson, J. L. *Natural production monitoring and evaluation*. (Idaho Department of Fish & Game, 2002).

71. Zimmerman, M. S., Kinsel, C., Beamer, E., Connor, E. J. & Pflug, D. E. Abundance, survival, and life history strategies of juvenile Chinook Salmon in the Skagit River, Washington. *Trans. Am. Fish. Soc.* **144,** 627–641 (2015).

72. McPherson, S. *et al.* Stock status and escapement goals for Chinook salmon stocks in Southeast Alaska. *Alsk. Dep. Fish Game Spec. Publ.* 03–01 (2003).

73. Seiler, D. *et al.* 2003 Juvenile Salmonid Production Evaluation Report Green River, Wenatchee River, and Cedar Creek. (2004).

74. Carmichael, R. W., Hoffnagle, T., Feldhaus, J., Eddy, D. & Albrecht, N. *Upper Grande Ronde River Spring Chinook Salmon Hatchery Program Review*. 20 (Oregon Department of Fish and Wildlife).

75. Kinsel, C., Zimmerman, M., Kishimoto, L. & Topping, P. Annual report: 2007 Skagit River salmon production evaluation. *Wash. Dep. Fish Wildl. Olymp.* (2008).

76. Kiyohara, K. & Volkhardt, G. *Evaluation of downstream migrant salmon production in 2007 from the Cedar River and Bear Creek*. (Washington Department of Fish and Wildlife, Fish Program, Science Division, 2008).

77. Lamperth, J., Zimmerman, M. S., Claiborne, A. M., Campbell, L. & Hildebrandt, A. Evaluation of Coweeman River salmonids in 2012 and 2013: juvenile production and other activities. *Wash. Dep. Fish Wildl. FPA* 14–03 (2014).

78. Lamperth, J. *et al.* Coweeman River salmonid production evaluation: 2011 completion report. *Wash. Dep. Fish Wildl. FPA* 13–01 (2013).

79. Seiler, D., Volkhardt, G., Fleischer, L. & Kiyohara, K. *Evaluation of downstream migrant salmon production in 2001 from the Cedar River and Bear Creek*. (Washington Department of Fish and Wildlife, Fish Program, Science Division, 2005).

80. ADF&G. Information by Fishery.

81. O�Neill, S. M., Ylitalo, G. M. & West, J. E. Energy content of Pacific salmon as prey of northern and southern resident killer whales. *Endanger. Species Res.* **25,** 265–28 (2014).

82. Teel, D. J., Burke, B. J., Kuligowski, D. R., Morgan, C. A. & Van Doornik, D. M. Genetic identification of Chinook Salmon: stock-specific distributions of juveniles along the Washington and Oregon coasts. *Mar. Coast. Fish.* **7,** 274–300 (2015).

83. Weitkamp, L. A. *et al.* Stock-specific size and timing at ocean entry of Columbia River juvenile Chinook salmon and steelhead: implications for early ocean growth. *Mar. Coast. Fish.* (2015).

84. Clemons, E. *et al.* FISHERY REGULATION ASSESSMENT MODEL (FRAM). (2006).

85. Weitkamp, L. A. Marine distributions of Chinook salmon from the west coast of North America determined by coded wire tag recoveries. *Trans. Am. Fish. Soc.* **139,** 147–170 (2010).
